# Supplementary material for: Artificial sweeteners and cancer risk: Results from the NutriNet-Santé population-based cohort study
Source: PLoS Med. 2022 Mar 24;19(3):e1003950. doi: 10.1371/journal.pmed.1003950 (PMC8946744; doi:10.1371/journal.pmed.1003950)
Supplement: S1 Appendix — Method A: Methodology for identification of energy under-reporting and validation studies for the 24-hour web-based dietary records. Fig A: Proportional hazards assumption testing using rescaled Schoenfeld-type residuals for the association between total artificial sweetener intake and cancer risk, NutriNet-Santé cohort, France, 2009–2021 (n = 102,865). Method B: Multiple imputation by chained equations. Table A: Number of participants in each combination of artificial sweetener consumption for aspartame, acesulfame-K, and sucralose (non-consumers, consumers of 1 type of artificial sweetener, consumers of 2 types, and consumers of 3 types), NutriNet-Santé cohort, France, 2009–2021 (n = 102,865). Table B: Associations between total artificial sweetener, acesulfame-K, aspartame, and sucralose intakes (mg/day) and breast cancer risk in premenopausal and postmenopausal women, NutriNet-Santé cohort, France, 2009–2021 (n = 80,711). Fig B: Forest plots presenting the minimally adjusted and fully adjusted associations between total artificial sweetener, aspartame, acesulfame-K, and sucralose intakes (mg/day) and cancer risk, NutriNet-Santé cohort, France, 2009–2021 (n = 102,865). Result A: Results from competing risk analyses (cause-specific Cox proportional hazards models). Table C: Association between total artificial sweetener, aspartame, acesulfame-K, and sucralose intakes (mg/day) and cancer risk, NutriNet-Santé cohort, France, 2009–2021 (n = 102,865)—consumers versus non-consumers. Table D: Interaction tests for BMI and between the 3 main artificial sweeteners (aspartame, acesulfame-K, and sucralose) for all studied outcomes. Fig C: Cancer risk associated with combined artificial sweetener and sugar intakes, NutriNet-Santé cohort, France, 2009–2021 (n = 102,865). Table E: Overall cancer risk associated with combined artificial sweetener and sugar intakes: 2-by-2 comparisons across categories, NutriNet-Santé cohort, France, 2009–2021 (n = 102,865). Table F: Focus on th [file pmed.1003950.s002.docx]

Online Supplementary Material

Artificial sweeteners and cancer risk: results from the NutriNet-Santé population-based cohort study

Charlotte Debras^1,2*^, Eloi Chazelas^1,2^, Bernard Srour^1,2^, Nathalie Druesne-Pecollo^1,2^, Younes Esseddik^1^, Fabien Szabo de Edelenyi^1^, Cédric Agaësse^1^, Alexandre De Sa^1^, Rebecca Lutchia^1^, Stéphane Gigandet^4^, Inge Huybrechts^2,5^, Chantal Julia^1,3^, Emmanuelle Kesse-Guyot^1,2^, Benjamin Allès^1^, Valentina A Andreeva^1^, Pilar Galan^1,2^, Serge Hercberg^1,2,3^, Mélanie Deschasaux-Tanguy^1,2^, Mathilde Touvier^1,2^

[*c.debras@eren.smbh.univ-paris13.fr](mailto:*c.debras@eren.smbh.univ-paris13.fr)

## Method A: Methodology for identification of under-energy reporting and validation studies for the 24h web-based dietary records

## Energy under-reporting was identified using Black’s method (1,2) based on the original method developed by Goldberg et al. (3), relying on the hypothesis that energy expenditure and intake, when weight is stable, are equal. Black’s equations are based on an estimate of the person’s basal metabolic rate (BMR) calculated via Schofield’s equations (4) and taking into account sex, age, height and weight, as well as physical activity level (PAL), number of 24h records, intra-individual variabilities of reported energy intake and BMR, and intra/intervariabilities of PAL. In the present study, intra-individual coefficients of variations for BMR and PAL were fixed using the values proposed by Black et al., i.e. 8.5 % and 15%, respectively. For identifying under-reporters, the 1.55 value of PAL was used. It corresponds to the WHO value for “light” activity, which is the probable minimum energy requirement for a normally active but sedentary individual (not sick, disabled or frail elderly). A higher value might have exaggerated the extent of under-reporting. Some under-reporting individuals were not excluded if their reported energy intake, initially estimated abnormally low, was found to be likely in case of recent weight variation or reported practice of weight-loss restrictive diet or proactive statement of the participant that he/she ate less than usual on the day of the dietary record. In this study 18,818 participants (corresponding to 14.7% of the subjects) were considered as under-energy reporters and were excluded from the study. This proportion of under-reporters is common, for instance in the nationally representative INCA 3 study conducted in 2016 by the French Food Safety Agency (5) 18% of adults participants were identified as under-reporters using the Black method.

**Figure A:** Proportional hazard assumption testing using rescaled Schoenfeld-type residuals for the association between total artificial sweetener intakes and cancer risk, NutriNet-Santé cohort, France, 2009-2021 (n =102,865)


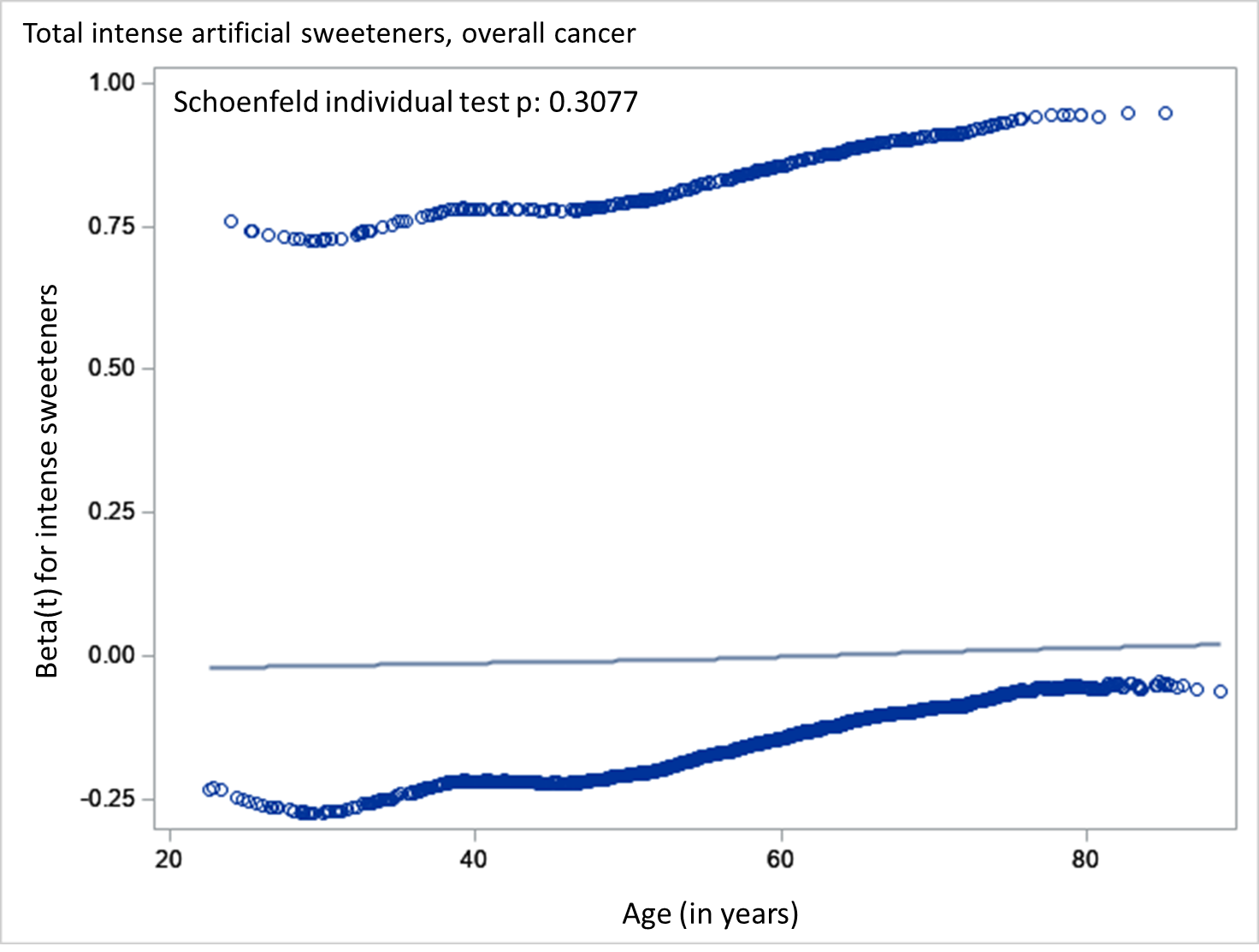


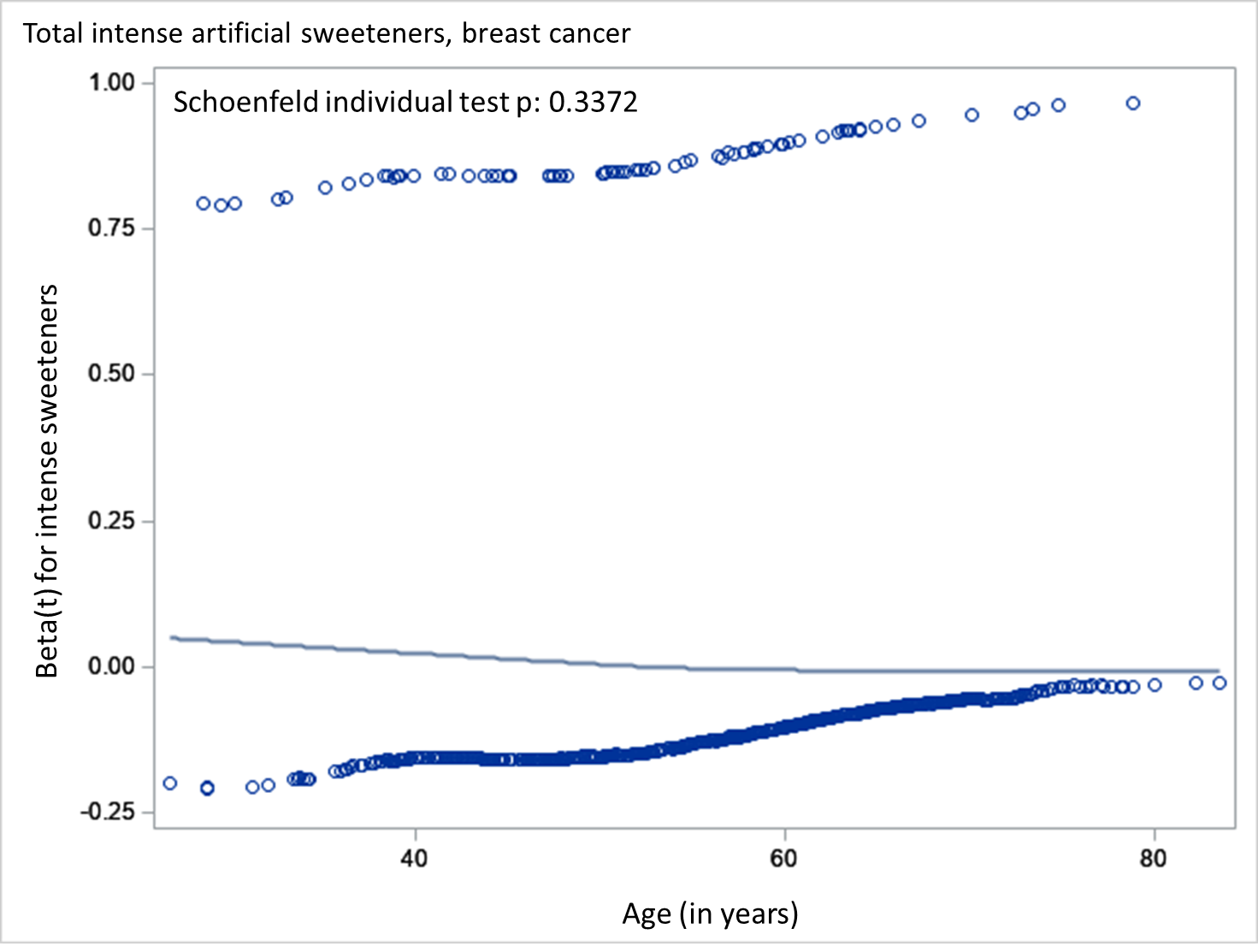


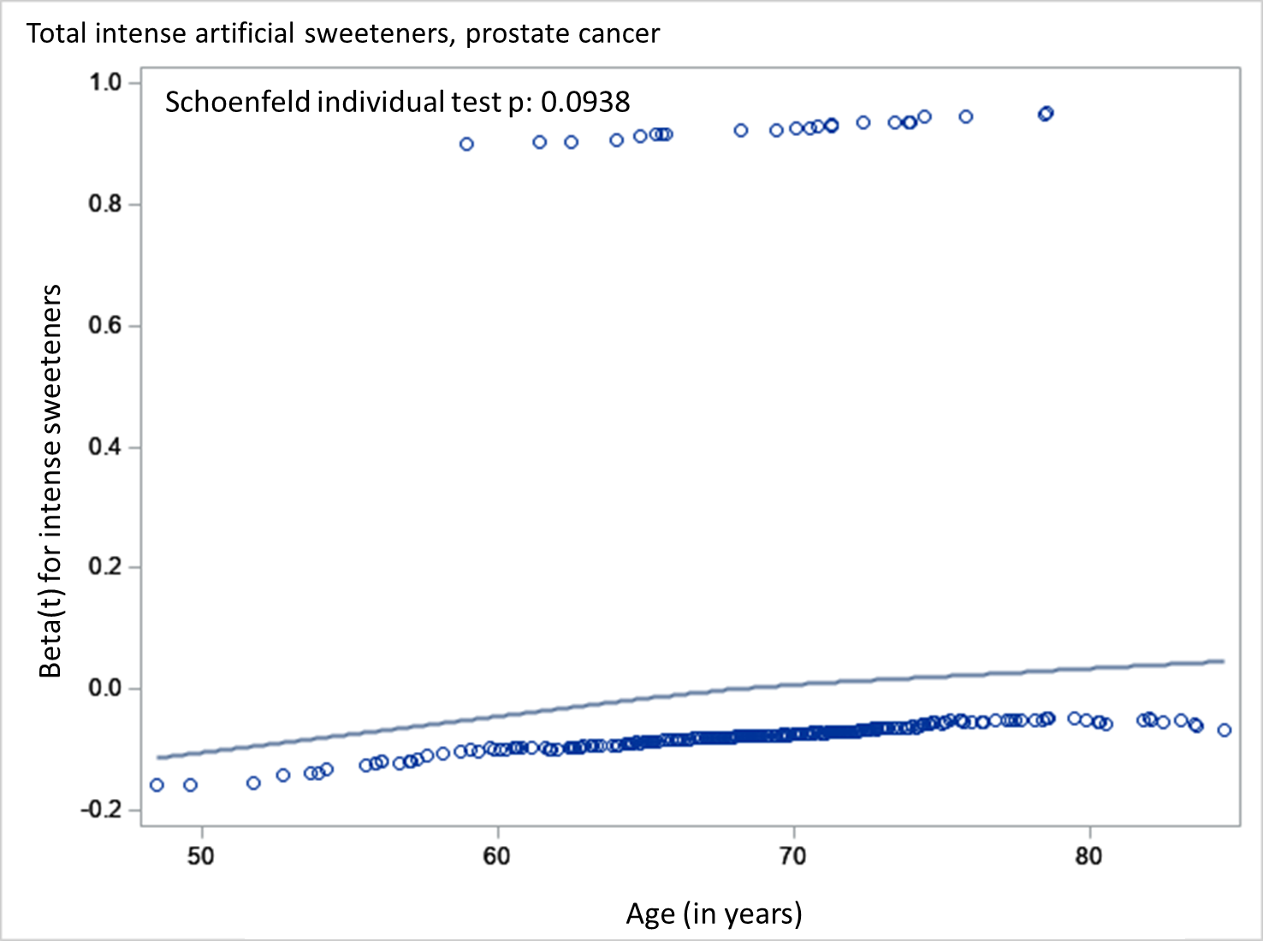


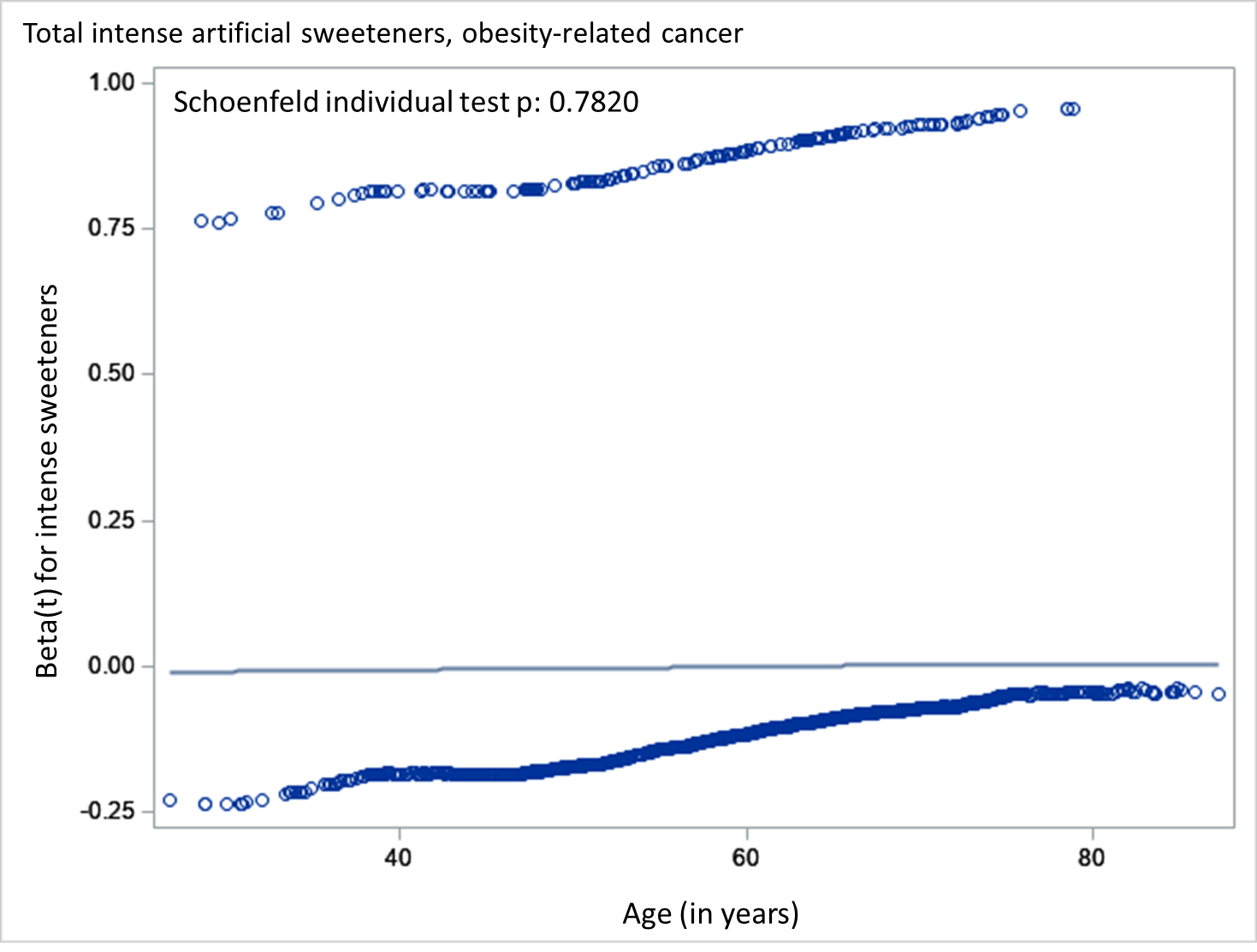


## Method B: Multiple Imputation by Chained Equations

Missing values for covariates were handled using the Multiple Imputation by Chained Equations (MICE) method (6) (15 imputed datasets) for the following covariates: smoking status (0.13% of missing data), level of education (0.79%), physical activity (13.89%), prevalence of diabetes (0.50%), BMI (2.90%), age at first child (<0.01%), age at menarche (0.73%), number of biological children (<0.01%), menopausal status (0.55%), age at menopause (0.02%) and hormonal treatment for menopause (0.73%).

**Table A:** Number of participants in each combination of artificial sweetener consumption for aspartame, acesulfame-K and sucralose (non-consumers, consumers of one type of artificial sweetener, consumers of two types, consumers of three types), NutriNet-Santé cohort, France, 2009-2021 (*n* =102,865)

|  | *n* | % |
| --- | --- | --- |
| No aspartame, acesulfame-K or sucralose | 67,074 | 65.21 |
| Aspartame only | 117 | 0.11 |
| Acesulfame-K only | 365 | 0.35 |
| Sucralose only | 468 | 0.45 |
| Aspartame + Acesulfame-K | 21,309 | 20.72 |
| Aspartame + Sucralose | 0 | 0 |
| Acesulfame-K + Sucralose | 6260 | 6.09 |
| Aspartame + Acesulfame-K + Sucralose | 7272 | 7.07 |

**Table B:** Associations between total artificial sweeteners, acesulfame-K, aspartame and sucralose intakes (mg/d) and breast cancer risk in premenopausal and postmenopausal women, NutriNet-Santé cohort, France, 2009-2021 (*n* = 80,711)^1^

|  | Exposure (mg/d) |  | Non-consumers | Consumers | *P* | Non-consumers | Lower-consumers^2^ | Higher-consumers^2^ | *P*-trend |
| --- | --- | --- | --- | --- | --- | --- | --- | --- | --- |
| Premenopausal breast cancer | Total artificial sweeteners | Participants / Incident cases | 36,909 / 221 | 24,433 / 174 |  | 36,909 / 221 | 12,216 / 91 | 12,217 / 83 |  |
|  |  | HR (95% CI) | 1 | 1.12 (0.91 to 1.38) | 0.299 | 1 | 1.09 (0.84 to 1.40) | 1.15 (0.89 to 1.50) | 0.267 |
|  | Aspartame | Participants / Incident cases | 42,721 / 261 | 18,621 / 134 |  | 42,721 / 261 | 9,321 / 73 | 9,300 / 61 |  |
|  |  | HR (95% CI) | 1 | 1.08 (0.86 to 1.35) | 0.507 | 1 | 1.08 (0.83 to 1.42) | 1.07 (0.79 to 1.46) | 0.564 |
|  | Acesulfame-K | Participants / Incident cases | 38,733 / 231 | 22,609 / 164 |  | 38,733 / 231 | 11,304 / 97 | 11,305 / 67 |  |
|  |  | HR (95% CI) | 1 | 1.14 (0.91 to 1.43) | 0.255 | 1 | 1.17 (0.91 to 1.51) | 1.07 (0.78 to 1.48) | 0.441 |
|  | Sucralose | Participants / Incident cases | 52,333 / 320 | 9,009 / 75 |  | 52,333 / 320 | 4,565 / 45 | 4,444 / 30 |  |
|  |  | HR (95% CI) | 1 | 1.26 (0.97 to 1.64) | 0.089 | 1 | 1.30 (0.94 to 1.79) | 1.20 (0.81 to 1.78) | 0.144 |
| Postmenopausal breast cancer | Total artificial sweeteners | Participants / Incident cases | 18,019 / 335 | 10,675 / 249 |  | 18,019 / 335 | 5,337 / 125 | 5,338 / 124 |  |
|  |  | HR (95% CI) | 1 | 1.14 (0.96 to 1.36) | 0.126 | 1 | 1.09 (0.89 to 1.35) | 1.20 (0.97 to 1.50) | 0.087 |
|  | Aspartame | Participants / Incident cases | 20,405 / 386 | 8,289 / 198 |  | 20,405 / 386 | 4,144 / 100 | 4,145 / 98 |  |
|  |  | HR (95% CI) | 1 | 1.18 (0.98 to 1.42) | 0.075 | 1 | 1.13 (0.91 to 1.42) | 1.24 (0.98 to 1.57) | 0.060 |
|  | Acesulfame-K | Participants / Incident cases | 18,610 / 350 | 10,084 / 234 |  | 18,610 / 350 | 5,042 / 120 | 5,042 / 114 |  |
|  |  | HR (95% CI) | 1 | 1.12 (0.93 to 1.34) | 0.241 | 1 | 1.09 (0.88 to 1.35) | 1.15 (0.91 to 1.47) | 0.211 |
|  | Sucralose | Participants / Incident cases | 24,725 / 506 | 3,969 / 78 |  | 24,725 / 506 | 1,984 / 39 | 1,985 / 39 |  |
|  |  | HR (95% CI) | 1 | 0.82 (0.64 to 1.06) | 0.126 | 1 | 0.78 (0.56 to 1.08) | 0.88 (0.63 to 1.23) | 0.209 |

^1^Women contributed person time to the premenopausal breast cancer model until their age at menopause and to the postmenopausal breast cancer model from their age at menopause.

Median follow-up times for premenopausal breast and postmenopausal breast cancers were, respectively, 7.4 and 8.6 y. Person-years were, respectively, 403,869 and 228,372.

Multivariable Cox proportional hazard models were adjusted for (= main model) age (time-scale), sex (except for breast and prostate), BMI (continuous, kg/m^2^), height (continuous, cm), percentage of weight gain during follow-up (continuous), physical activity (categorical International Physical Activity Questionnaire variable: high, moderate, low, missing value), smoking status (categorical: never, former, current smokers), number of smoked cigarettes in pack-years (continuous), educational level (categorical: less than high school degree, <2 y after high school degree, ≥2 y after high school degree), number of 24h dietary records (continuous), family history of cancer (categorical: yes, no), prevalent diabetes (categorical: yes, no), energy intake without alcohol (continuous variable : kcal/d), alcohol intake (continuous, g/d), sodium (continuous, g/d), saturated fatty acids (continuous, g/d), fibre intake (continuous, g/d), sugar intake (continuous, g/d), fruit and vegetable intake (continuous, g/d), whole food intake (continuous, g/d) and dairy product intake (continuous, g/d). Breast cancer model was also adjusted for age at menarche (categorical: <12 y old, ≥12 y old), age at first child (categorical: no child, before 30 y, ≥30 y), number of biological children (continuous), baseline menopausal status (categorical: menopausal, non-menopausal), oral contraceptive use at baseline and during follow-up (categorical: yes, no), and hormonal treatment for menopause at baseline and during follow-up (categorical: yes, no).

Breast cancer analyses were additionally adjusted for: age at menarche (categorical: <12 y old, ≥12 y old), age at first child (categorical: no child, before 30 y, ≥30 y), number of biological children (continuous), baseline menopausal status (categorical: menopausal, non-menopausal), oral contraceptive use at baseline and during follow-up (categorical: yes, no), hormonal treatment for menopause at baseline and during follow up (categorical: yes, no).

In addition, all models were mutually adjusted for artificial sweetener intake other than the one studied.

^2^Sex specific cut-offs among consumers of artificial sweeteners were 19.00 mg/d in women (20.17 and 15.43 mg/d, among premenopausal and postmenopausal women, respectively).

Sex specific cut-offs among consumers of aspartame were 15.39 mg/d in women (16.44 and 12.16 mg/d, among premenopausal and postmenopausal women, respectively).

Sex specific cut-offs among consumers of acesulfame-K were 5.50 mg/d in women (6.71 and 3.57 mg/d, among premenopausal and postmenopausal women, respectively).

Sex specific cut-offs among consumers of sucralose were 3.43 mg/d in women (3.44 and 2.71 mg/d, among premenopausal and postmenopausal women, respectively).

During premenopausal women's follow-up, 140 competing deaths occurred and 730 competing cases of cancers other than premenopausal breast cancers were diagnosed. Cause-specific HRs (95% CIs) for death in the third tertile of artificial sweeteners, acesulfame-K, aspartame and sucralose intakes compared with non-consumers were, respectively, 0.69 (0.43 to 1.11), P-trend = 0.1, 0.81 (0.49 to 1.32), P-trend = 0.3, 0.84 (0.52 to 1.36), P-trend = 0.4, and 0.82 (0.40 to 1.70), P-trend = 0.8. Cause-specific HRs (95% CIs) for all cancers except premenopausal breast in the third tertile of artificial sweeteners, acesulfame-K, aspartame and sucralose intakes compared with non-consumers were, respectively, 1.02 (0.83 to 1.25), P-trend = 0.5, 1.05 (0.85 to 1.30), P-trend = 0.4, 1.11 (0.89 to 1.37), P-trend = 0.2, and 0.84 (0.62 to 1.15), P-trend = 0.3.

P for Heterogeneity tests between pre and postmenopausal models = 0.440 for total artificial sweeteners, 0.332 for aspartame, 0.359 for acesulfame-K and 0.015 for sucralose.

**Figure B**: Forest plots presenting the minimally-adjusted^1^ and fully adjusted^2^ associations between total artificial sweeteners, aspartame, acesulfame-K and sucralose intakes (mg/d) and cancer risk, NutriNet-Santé cohort, France, 2009-2021 (*n* = 102,865)


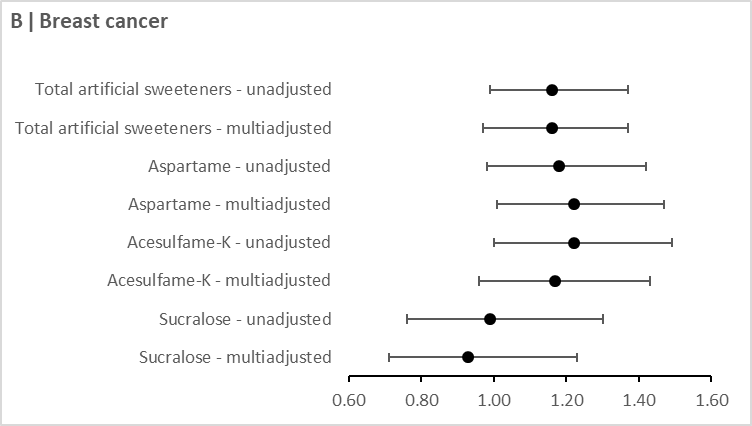

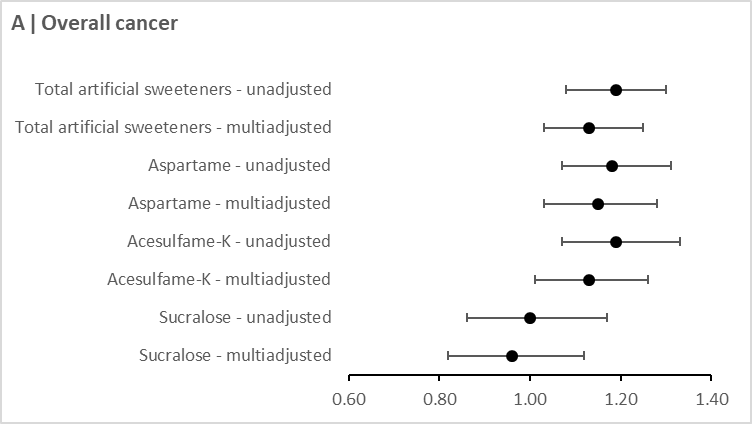

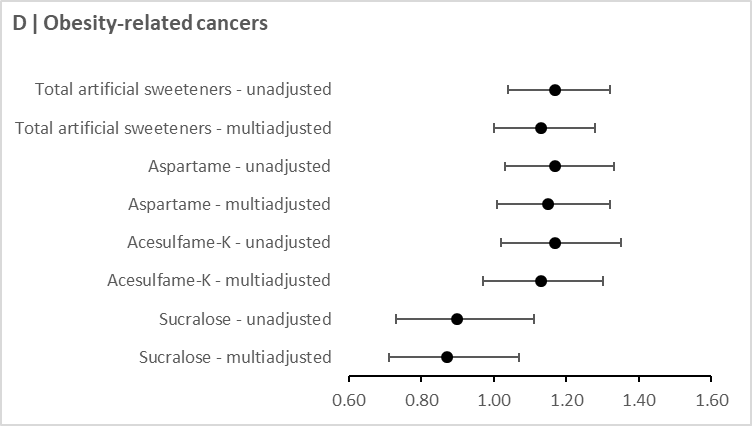

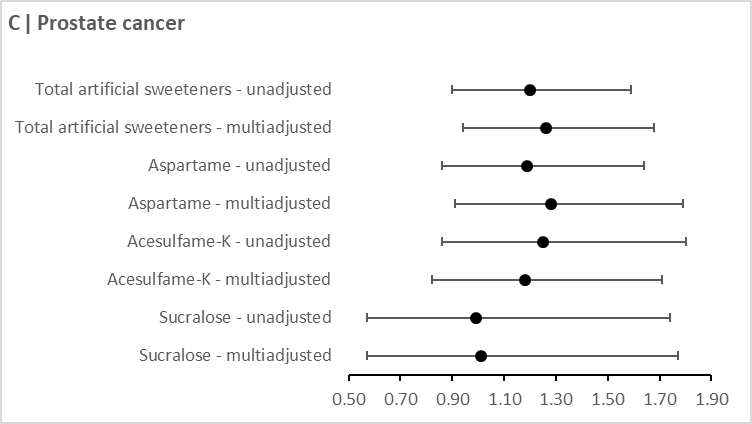


^1^ Adjusted for age (time-scale) and sex only.

^2^ Adjusted for age (time-scale), sex (except for breast and prostate), BMI, height, weight gain during follow-up, physical activity, smoking status, number of smoked cigarettes in pack-years, educational level, number of 24h dietary records, family history of cancer, prevalent diabetes, energy intake without alcohol, alcohol, sodium, saturated fatty acids, fibre, sugar, fruit and vegetable, whole-grain foods, dairy products. All models were mutually adjusted for artificial sweetener intake other than the one studied. Breast cancer models were also adjusted for age at menarche, age at first child, number of biological children, baseline menopausal status, oral contraceptive use at baseline and during follow-up, and hormonal treatment for menopause at baseline and during follow-up.

## Result A: Results from competing risk analyses (cause-specific Cox proportional hazard models)

During follow-up, 463 competing deaths occurred, 1,804 cancer cases of than breast and 2,230 cancer cases other than prostate were diagnosed. Cause-specific HRs (95% CIs) for death in the highest category of artificial sweeteners, aspartame, acesulfame-K and sucralose intakes compared with non-consumers were, respectively, 1.09 (0.85 to 1.39), P-trend = 0.4, 1.15 (0.88 to 1.49), P-trend = 0.09, 1.02 (0.76 to 1.37), P-trend = 0.4, and 0.92 (0.61 to 1.40), P-trend = 0.6. Cause-specific HRs (95% CIs) for all cancers except breast in the highest category of artificial sweeteners, aspartame, acesulfame-K and sucralose intakes compared with non-consumers were, respectively, 1.06 (0.93 to 1.22), P-trend = 0.09, 1.10 (0.95 to 1.27), P-trend = 0.04, 1.09 (0.94 to 1.26), P-trend = 0.08, and 0.91 (0.73 to 1.13), P-trend = 0.6. Cause-specific HRs (95% CIs) for all cancers except prostate in the highest category of artificial sweeteners, aspartame, acesulfame-K and sucralose intakes compared with non-consumers were, respectively, 1.11 (0.99 to 1.25), P-trend = 0.008, 1.16 (1.02 to 1.31), P-trend = 0.002, 1.15 (1.02 to 1.31), P-trend = 0.005, and 0.95 (0.79 to 1.14), P-trend = 1.0. Cause-specific HRs (95% CIs) for all cancers except obesity-related cancers in the highest category of artificial sweeteners, aspartame, acesulfame-K and sucralose intakes compared with non-consumers were, respectively, 1.13 (0.97 to 1.32), P-trend = 0.02, 1.16 (0.99 to 1.37), P-trend = 0.02, 1.15 (0.98 to 1.36), P-trend = 0.02, and 1.12 (0.90 to 1.40), P-trend = 0.2. During postmenopausal women's follow-up, 283 competing deaths occurred and 848 competing cases of cancers other than postmenopausal breast cancers were diagnosed. Cause-specific HRs (95% CIs) for death in the third tertile of artificial sweeteners, acesulfame-K, aspartame and sucralose intakes compared with non-consumers were, respectively, 1.09 (0.80 to 1.47), P-trend = 0.6, 1.24 (0.90 to 1.70), P-trend = 0.2, 1.20 (0.86 to 1.66), P-trend = 0.1, and 1.04 (0.66 to 1.66), P-trend = 0.8. Cause-specific HRs (95% CIs) for all cancers except postmenopausal breast in the third tertile of artificial sweeteners, acesulfame-K, aspartame and sucralose intakes compared with non-consumers were, respectively, 0.99 (0.82 to 1.19), P-trend = 0.9, 1.09 (0.91 to 1.32), P-trend = 0.5, 1.02 (0.83 to 1.26), P-trend = 0.6, and 0.95 (0.72 to 1.25), P-trend = 0.6.

**Table C:** Association between total artificial sweeteners, aspartame, acesulfame-K and sucralose intakes (mg/d) and cancer risk, NutriNet-Santé cohort, France, 2009-2021 (*n* = 102,865) – Consumers versus non-consumers^1^

| Cancer site | Exposure (mg/d) |  | Non-consumers | Consumers | *P*-trend |
| --- | --- | --- | --- | --- | --- |
| All cancers | Total artificial sweeteners | Participants / Incident cases | 64,901 / 2013 | 37,976 / 1345 |  |
|  |  | HR (95% CI) | 1 | 1.14 (1.06 to 1.22) | <0.001 |
|  | Aspartame | Participants / Incident cases | 74,179 / 2309 | 28,698 / 1049 |  |
|  |  | HR (95% CI) | 1 | 1.13 (1.05 to 1.23) | 0.001 |
|  | Acesulfame-K | Participants / Incident cases | 67,671 / 2096 | 35,206 / 1262 |  |
|  |  | HR (95% CI) | 1 | 1.12 (1.04 to 1.22) | 0.003 |
|  | Sucralose | Participants / Incident cases | 88,877 / 2883 | 14,000 / 475 |  |
|  |  | HR (95% CI) | 1 | 1.00 (0.91 to 1.11) | 0.926 |
| Breast cancer | Total artificial sweeteners | Participants / Incident cases | 49,358 / 556 | 31,363 / 423 |  |
|  |  | HR (95% CI) | 1 | 1.16 (0.94 to 1.43) | 0.156 |
|  | Aspartame | Participants / Incident cases | 56,721 / 647 | 24,000 / 332 |  |
|  |  | HR (95% CI) | 1 | 1.23 (0.99 to 1.53) | 0.061 |
|  | Acesulfame-K | Participants / Incident cases | 51,563 / 581 | 29,158 / 398 |  |
|  |  | HR (95% CI) | 1 | 1.11 (0.89 to 1.38) | 0.338 |
|  | Sucralose | Participants / Incident cases | 69,198 / 826 | 11,523 / 153 |  |
|  |  | HR (95% CI) | 1 | 0.85 (0.63 to 1.14) | 0.267 |
| Prostate cancer | Total artificial sweeteners | Participants / Incident cases | 15,543 / 282 | 6,613 / 121 |  |
|  |  | HR (95% CI) | 1 | 1.05 (0.85 to 1.31) | 0.641 |
|  | Aspartame | Participants / Incident cases | 17,458 / 310 | 4,698 / 93 |  |
|  |  | HR (95% CI) | 1 | 1.07 (0.84 to 1.37) | 0.562 |
|  | Acesulfame-K | Participants / Incident cases | 16,108 / 288 | 6,048 / 115 |  |
|  |  | HR (95% CI) | 1 | 1.09 (0.86 to 1.39) | 0.460 |
|  | Sucralose | Participants / Incident cases | 19,679 / 365 | 2,477 / 38 |  |
|  |  | HR (95% CI) | 1 | 0.91 (0.64 to 1.27) | 0.572 |
| Obesity-related cancers | Total artificial sweeteners | Participants / Incident cases | 64,901 / 1232 | 37,976 / 791 |  |
|  |  | HR (95% CI) | 1 | 1.10 (1.01 to 1.21) | 0.038 |
|  | Aspartame | Participants / Incident cases | 74,179 / 1401 | 28,698 / 622 |  |
|  |  | HR (95% CI) | 1 | 1.11 (1.01 to 1.23) | 0.036 |
|  | Acesulfame-K | Participants / Incident cases | 67,671 / 1275 | 35,206 / 748 |  |
|  |  | HR (95% CI) | 1 | 1.10 (0.99 to 1.22) | 0.064 |
|  | Sucralose | Participants / Incident cases | 88,877 / 1756 | 14000 / 267 |  |
|  |  | HR (95% CI) | 1 | 0.94 (0.82 to 1.07) | 0.367 |

^1^Median follow-up times for overall, breast, prostate and obesity-related cancers were, respectively, 7.7, 7.6, 8.0 and 7.7 y. Person-years were, respectively 708,905, 551,803, 157,102 and 708,905. Multivariable Cox proportional hazard models were adjusted for (= main model) age (time-scale), sex (except for breast and prostate), BMI (continuous, kg/m2), height (continuous, cm), percentage of weight gain during follow-up (continuous), physical activity (categorical International Physical Activity Questionnaire variable: high, moderate, low, missing value), smoking status (categorical: never, former, current smokers), number of smoked cigarettes in pack-years (continuous), educational level (categorical: less than high school degree, <2 y after high school degree, ≥2 y after high school degree), number of 24h dietary records (continuous), family history of cancer (categorical: yes, no), prevalent diabetes (categorical: yes, no), energy intake without alcohol (continuous variable : kcal/d), daily intakes (continuous, g/d) of: alcohol, sodium, saturated fatty acids, fibre, sugar, fruit and vegetable, whole-grain foods and dairy products. Breast cancer models were also adjusted for age at menarche (categorical: <12 y old, ≥12 y old), age at first child (categorical: no child, before 30 y, ≥30 y), number of biological children (continuous), baseline menopausal status (categorical: menopausal, non-menopausal), oral contraceptive use at baseline and during follow-up (categorical: yes, no), and hormonal treatment for menopause at baseline and during follow-up (categorical: yes, no).

In addition, all models were mutually adjusted for artificial sweetener intake other than the one studied.

**Table D:** Interaction tests for BMI and between the three main artificial sweeteners (aspartame, acesulfame-K and sucralose) for all studied outcomes

|  | Outcome | P-interaction | |  |
| --- | --- | --- | --- | --- |
| **Interaction between BMI (</≥25 kg/m2) and each artificial sweetener** | | |  |  |
| BMI * total artificial sweeteners | Overall cancer | 0.908 | |  |
|  | Breast cancer | 0.700 | |  |
|  | Prostate cancer | 0.649 | |  |
|  | Obesity-related cancers | 0.577 | |  |
| BMI * aspartame | Overall cancer | 0.893 | |  |
|  | Breast cancer | 0.796 | |  |
|  | Prostate cancer | 0.884 | |  |
|  | Obesity-related cancers | 0.925 | |  |
| BMI * acesulfame-K | Overall cancer | 0.651 | |  |
|  | Breast cancer | 0.469 | |  |
|  | Prostate cancer | 0.311 | |  |
|  | Obesity-related cancers | 0.729 | |  |
| BMI * sucralose | Overall cancer | 0.773 | |  |
|  | Breast cancer | 0.763 | |  |
|  | Prostate cancer | 0.079 | |  |
|  | Obesity-related cancers | 0.842 | |  |
| **3-way interactions between each main artificial sweetener** | |  | | |
| Aspartame * acesulfame-K * sucralose | Overall cancer | 0.615 | |  |
|  | Breast cancer | 0.360 | |  |
|  | Prostate cancer | 0.784 | |  |
|  | Obesity-related cancers | 0.295 | |  |


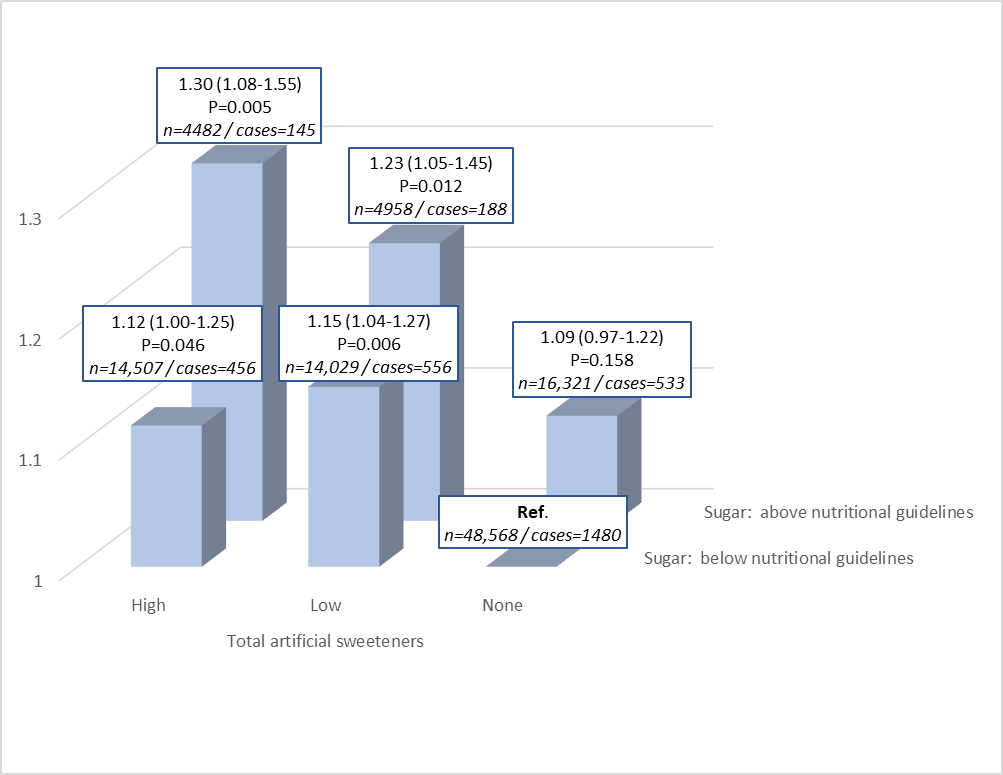
**Figure C:** Cancer risk associated with the combined exposure to artificial sweetener and sugar intakes, NutriNet -Santé cohort, France, 2009-2021 (n=102,865)

**Table E**: Overall cancer risk associated with combined exposure to artificial sweeteners and sugar intakes: two-by-two comparisons across categories, NutriNet -Santé cohort, France, 2009-2021 (n=102,865)

| **Reference** | **No artificial sweetener / Low sugar** | **Low artificial sweetener / Low sugar** | **High artificial sweetener / Low sugar** | **No artificial sweetener / High sugar** | **Low artificial sweetener / High sugar** | **High artificial sweetener / High sugar** |
| --- | --- | --- | --- | --- | --- | --- |
| **No artificial sweetener / Low sugar** | - | 1.15 (1.04-1.27) P=0.006 | 1.12 (1.00-1.25) P=0.046 | 1.09 (0.97-1.22) P=0.158 | 1.23 (1.05-1.45) P=0.012 | 1.30 (1.08-1.55) P=0.005 |
| **Low artificial sweetener / Low sugar** | 0.87 (0.79-0.96) P=0.006 | - | 0.97 (0.86-1.10) P=0.661 | 0.95 (0.83-1.08) P=0.424 | 1.07 (0.90-1.27) P=0.446 | 1.13 (0.93-1.37) P=0.221 |
| **High artificial sweetener / Low sugar** | 0.90 (0.80-1.00) P=0.046 | 1.03 (0.91-1.17) P=0.661 | - | 0.97 (0.84-1.12) P=0.712 | 1.10 (0.92-1.32) P=0.300 | 1.16 (0.95-1.41) P=0.139 |
| **No artificial sweetener / High sugar** | 0.92 (0.82-1.03) P=0.158 | 1.06 (1.92-1.21) P=0.424 | 1.03 (0.89-1.19) P=0.712 | - | 1.13 (0.96-1.34) P=0.149 | 1.19 (0.99-1.44) P=0.064 |
| **Low artificial sweetener / High sugar** | 0.81 (0.69-0.96) P=0.012 | 0.93 (0.79-1.11) P=0.446 | 0.91 (0.76-1.09) P=0.300 | 0.88 (0.75-1.05) P=0.149 | - | 1.05 (0.85-1.31) P=0.636 |
| **High artificial sweetener / High sugar** | 0.77 (0.64-0.93) P=0.005 | 0.89 (0.73-1.08) P=0.221 | 0.86 (0.71-1.05) P=1.139 | 0.84 (0.70-1.01) P=0.064 | 0.95 (0.76-1.18) P=0.636 | - |

**Table F:** Focus on the comparisons of cancer risk for participants with higher artificial sweetener consumption / lower sugar intakes versus participants with no artificial sweetener consumption / higher sugar intakes, NutriNet-Santé cohort, France, 2009-2021 (*n* = 102,865)^1^

| Cancer site | Exposure (mg/d) |  | No artificial sweetener consumption / higher sugar intakes | Higher artificial sweetener consumption / lower sugar intakes | *P*-value² |
| --- | --- | --- | --- | --- | --- |
| All cancers | Total artificial sweeteners | Participants / Incident cases | 16,321 / 533 | 14,507 / 456 |  |
|  |  | HR (95% CI) | 1 | 1.03 (0.89 to 1.19) | 0.712 |
|  | Aspartame | Participants / Incident cases | 19,113 / 615 | 11,296 / 370 |  |
|  |  | HR (95% CI) | 1 | 1.05 (0.90 to 1.22) | 0.532 |
|  | Acesulfame-K | Participants / Incident cases | 17,104 / 554 | 13,153 / 368 |  |
|  |  | HR (95% CI) | 1 | 1.04 (0.89 to 1.21) | 0.670 |
|  | Sucralose | Participants / Incident cases | 21,603 / 732 | 4,813 / 130 |  |
|  |  | HR (95% CI) | 1 | 0.87 (0.71 to 1.28) | 0.837 |
| Breast cancer | Total artificial sweeteners | Participants / Incident cases | 10,161 / 129 | 12,551 / 155 |  |
|  |  | HR (95% CI) | 1 | 0.96 (0.74 to 1.63) | 0.781 |
|  | Aspartame | Participants / Incident cases | 12,000 / 146 | 9,815 / 127 |  |
|  |  | HR (95% CI) | 1 | 1.06 (0.80 to 1.39) | 0.695 |
|  | Acesulfame-K | Participants / Incident cases | 10,690 / 132 | 11,495 / 132 |  |
|  |  | HR (95% CI) | 1 | 1.01 (0.76 to 1.35) | 0.929 |
|  | Sucralose | Participants / Incident cases | 13,870 / 181 | 4,219 / 45 |  |
|  |  | HR (95% CI) | 1 | 0.80 (0.56 to 1.14) | 0.212 |
| Prostate cancer | Total artificial sweeteners | Participants / Incident cases | 6,160 / 106 | 1,956 / 37 |  |
|  |  | HR (95% CI) | 1 | 1.30 (0.86 to 1.97) | 0.218 |
|  | Aspartame | Participants / Incident cases | 7,113 / 118 | 1,481 / 29 |  |
|  |  | HR (95% CI) | 1 | 1.29 (0.82 to 2.04) | 0.270 |
|  | Acesulfame-K | Participants / Incident cases | 6,414 / 106 | 1,658 / 22 |  |
|  |  | HR (95% CI) | 1 | 1.18 (0.71 to 1.96) | 0.531 |
|  | Sucralose | Participants / Incident cases | 7,733 / 131 | 594 / 6 |  |
|  |  | HR (95% CI) | 1 | 0.92 (0.40 to 2.14) | 0.850 |
| Obesity-related cancers | Total artificial sweeteners | Participants / Incident cases | 16,321 / 329 | 14,507 / 276 |  |
|  |  | HR (95% CI) | 1 | 1.02 (0.85 to 1.23) | 0.829 |
|  | Aspartame | Participants / Incident cases | 19,113 / 288 | 11,296 / 222 |  |
|  |  | HR (95% CI) | 1 | 1.05 (0.87 to 1.27) | 0.615 |
|  | Acesulfame-K | Participants / Incident cases | 17,104 / 336 | 13,153 / 222 |  |
|  |  | HR (95% CI) | 1 | 1.05 (0.86 to 1.28) | 0.644 |
|  | Sucralose | Participants / Incident cases | 21,603 / 444 | 4,813 / 74 |  |
|  |  | HR (95% CI) | 1 | 0.83 (0.64 to 1.08) | 0.160 |

^1^Participants were cross-categorized into 6 classes according to their intake levels of artificial sweeteners/aspartame/acesulfame-K/sucralose (3 categories: non-, lower-, higher-consumers) and sugar (2 categories, adherence to nutritional guidelines of less than 100g/d of sugar, excluding lactose and galactose: yes, no).

²This table provides the results for the comparison between the categories “Higher artificial sweetener consumption / sugar intakes below the official recommended level” and “No artificial sweetener consumption / sugar intakes exceeding the recommended level (7)”=reference.

(7) The official French recommendation is intake below 100g/d of all types of simple sugars except lactose and galactose (i.e. saccharose, fructose, glucose, etc., including sugar from corn syrup, inverted sugar etc.)

Median follow-up times for overall, breast, prostate and obesity-related cancers were, respectively, 7.7, 7.6, 8.0 and 7.7 y. Person-years were, respectively 708,905, 551,803, 157,102 and 708,905. Multivariable Cox proportional hazard models were adjusted for (= main model) age (time-scale), sex (except for breast and prostate), BMI (continuous, kg/m2), height (continuous, cm), percentage of weight gain during follow-up (continuous), physical activity (categorical International Physical Activity Questionnaire variable: high, moderate, low, missing value), smoking status (categorical: never, former, current smokers), number of smoked cigarettes in pack-years (continuous), educational level (categorical: less than high school degree, <2 y after high school degree, ≥2 y after high school degree), number of 24h dietary records (continuous), family history of cancer (categorical: yes, no), prevalent diabetes (categorical: yes, no), energy intake without alcohol (continuous variable : kcal/d), daily intakes (continuous, g/d) of: alcohol, sodium, saturated fatty acids, fibre, fruit and vegetable, whole-grain foods and dairy products. Breast cancer models were also adjusted for age at menarche (categorical: <12 y old, ≥12 y old), age at first child (categorical: no child, before 30 y, ≥30 y), number of biological children (continuous), baseline menopausal status (categorical: menopausal, non-menopausal), oral contraceptive use at baseline and during follow-up (categorical: yes, no), and hormonal treatment for menopause at baseline and during follow-up (categorical: yes, no).

In addition, all models were mutually adjusted for artificial sweetener intake other than the one studied.

**Table G:** Association between artificial sweetener intake (mg/d) and cancer risk, NutriNet-Santé cohort, France, 2009-2021 (*n* = 102,865)^1^ – Sensitivity analyses

| Cancer site | Exposure (mg/d) | Model |  | Non-consumers | Lower-consumers^2^ | Higher-consumers^2^ | *P*-trend |
| --- | --- | --- | --- | --- | --- | --- | --- |
| All cancers | Total artificial sweeteners | Main | Participants / Incident cases | 64,892 / 2013 | 18,986 / 744 | 18,987 / 601 |  |
|  |  |  | HR (95% CI) | 1 | 1.14 (1.05 to 1.25) | 1.13 (1.03 to 1.25) | 0.002 |
|  |  | 1 | Participants / Incident cases | 32,914 / 1522 | 12,081 / 560 | 12,081 / 550 |  |
|  |  |  | HR (95% CI) | 1 | 1.11 (1.00 to 1.22) | 1.11 (1.00 to 1.23) | 0.020 |
|  |  | 2 | Participants / Incident cases | 64,055 / 1954 | 18,475 / 703 | 18,475 / 562 |  |
|  |  |  | HR (95% CI) | 1 | 1.14 (1.04 to 1.24) | 1.15 (1.04 to 1.26) | 0.001 |
|  |  | 3 | Participants / Incident cases | 64,892 / 2013 | 18,986 / 744 | 18,987 / 601 |  |
|  |  |  | HR (95% CI) | 1 | 1.14 (1.05 to 1.24) | 1.13 (1.02 to 1.24) | 0.002 |
|  |  | 4 | Participants / Incident cases | 64,892 / 2013 | 18,986 / 744 | 18,987 / 601 |  |
|  |  |  | HR (95% CI) | 1 | 1.14 (1.05 to 1.25) | 1.13 (1.03 to 1.24) | 0.002 |
|  |  | 5 | Participants / Incident cases | 64,892 / 2013 | 18,986 / 744 | 18,987 / 601 |  |
|  |  |  | HR (95% CI) | 1 | 1.15 (1.05 to 1.25) | 1.14 (1.03 to 1.25) | 0.001 |
|  |  | 6 | Participants / Incident cases | 64,892 / 2013 | 18,986 / 744 | 18,987 / 601 |  |
|  |  |  | HR (95% CI) | 1 | 1.14 (1.05 to 1.25) | 1.13 (1.02 to 1.24) | 0.003 |
|  |  | 7 | Participants / Incident cases | 64,892 / 2013 | 18,986 / 744 | 18,987 / 601 |  |
|  |  |  | HR (95% CI) | 1 | 1.15 (1.05 to 1.25) | 1.14 (1.03 to 1.25) | 0.001 |
|  |  | 8 | Participants / Incident cases | 83,882 / 2812 | 18,898 / 784 | 18,898 / 622 |  |
|  |  |  | HR (95% CI) | 1 | 1.13 (1.04 to 1.22) | 1.11 (1.02 to 1.22) | 0.003 |
|  |  | 9 | Participants / Incident cases | 64,892 / 2013 | 18,986 / 744 | 18,987 / 601 |  |
|  |  |  | HR (95% CI) | 1 | 1.13 (1.04 to 1.23) | 1.14 (1.04 to 1.26) | 0.001 |
|  |  | 10 | Participants / Incident cases | 64,892 / 2013 | 18,986 / 744 | 18,987 / 601 |  |
|  |  |  | HR (95% CI) | 1 | 1.14 (1.05 to 1.25) | 1.13 (1.03 to 1.25) | 0.002 |
|  |  | 11 | Participants / Incident cases | 58,243 / 1508 | 17,602 / 574 | 17,539 / 445 |  |
|  |  |  | HR (95% CI) | 1 | 1.17 (1.06 to 1.29) | 1.09 (0.97 to 1.21) | 0.028 |
|  |  | 12 | Participants / Incident cases | 53,713 / 1748 | 15,609 / 574 | 15,610 / 532 |  |
|  |  |  | HR (95% CI) | 1 | 1.13 (1.03 to 1.24) | 1.18 (1.06 to 1.30) | <0.001 |
|  | Aspartame | Main | Participants / Incident cases | 74,169 / 2309 | 14,345 / 572 | 14,351 / 477 |  |
|  |  |  | HR (95% CI) | 1 | 1.12 (1.02 to 1.23) | 1.15 (1.03 to 1.28) | 0.002 |
|  |  | 1 | Participants / Incident cases | 39,102 / 1784 | 8987 / 434 | 8987 / 414 |  |
|  |  |  | HR (95% CI) | 1 | 1.10 (0.99 to 1.22) | 1.06 (0.94 to 1.19) | 0.166 |
|  |  | 2 | Participants / Incident cases | 72,203 / 2238 | 13,893 / 542 | 13,909 / 439 |  |
|  |  |  | HR (95% CI) | 1 | 1.13 (1.03 to 1.24) | 1.16 (1.04 to 1.29) | 0.002 |
|  |  | 3 | Participants / Incident cases | 74,169 / 2309 | 14,345 / 572 | 14,351 / 477 |  |
|  |  |  | HR (95% CI) | 1 | 1.12 (1.02 to 1.23) | 1.14 (1.03 to 1.27) | 0.003 |
|  |  | 4 | Participants / Incident cases | 74,169 / 2309 | 14,345 / 572 | 14,351 / 477 |  |
|  |  |  | HR (95% CI) | 1 | 1.12 (1.02 to 1.23) | 1.15 (1.03 to 1.28) | 0.003 |
|  |  | 5 | Participants / Incident cases | 74,169 / 2309 | 14,345 / 572 | 14,351 / 477 |  |
|  |  |  | HR (95% CI) | 1 | 1.12 (1.02 to 1.23) | 1.15 (1.03 to 1.28) | 0.002 |
|  |  | 6 | Participants / Incident cases | 74,169 / 2309 | 14,345 / 572 | 14,351 / 477 |  |
|  |  |  | HR (95% CI) | 1 | 1.12 (1.02 to 1.23) | 1.15 (1.03 to 1.28) | 0.002 |
|  |  | 7 | Participants / Incident cases | 74,169 / 2309 | 14,345 / 572 | 14,351 / 477 |  |
|  |  |  | HR (95% CI) | 1 | 1.13 (1.02 to 1.24) | 1.16 (1.04 to 1.29) | 0.002 |
|  |  | 8 | Participants / Incident cases | 92,997 / 3132 | 14,340 / 589 | 14,341 / 497 |  |
|  |  |  | HR (95% CI) | 1 | 1.09 (1.00 to 1.19) | 1.14 (1.03 to 1.26) | 0.004 |
|  |  | 9 | Participants / Incident cases | 74,169 / 2309 | 14,345 / 572 | 14,351 / 477 |  |
|  |  |  | HR (95% CI) | 1 | 1.14 (1.05 to 1.25) | 1.13 (1.02 to 1.25) | 0.003 |
|  |  | 10 | Participants / Incident cases | 74,169 / 2309 | 14,345 / 572 | 14,351 / 477 |  |
|  |  |  | HR (95% CI) | 1 | 1.12 (1.02 to 1.23) | 1.15 (1.03 to 1.28) | 0.003 |
|  |  | 11 | Participants / Incident cases | 66,674 / 1729 | 13,355 / 443 | 13,355 / 355 |  |
|  |  |  | HR (95% CI) | 1 | 1.15 (1.04 to 1.29) | 1.11 (0.99 to 1.26) | 0.017 |
|  |  | 12 | Participants / Incident cases | 61,406 / 2005 | 11,772 / 489 | 11,754 / 414 |  |
|  |  |  | HR (95% CI) | 1 | 1.13 (1.02 to 1.25) | 1.17 (1.05 to 1.31) | 0.002 |
|  | Acesulfame-K | Main | Participants / Incident cases | 67,662 / 2096 | 17,601 / 766 | 17,602 / 496 |  |
|  |  |  | HR (95% CI) | 1 | 1.12 (1.03 to 1.22) | 1.13 (1.01 to 1.26) | 0.007 |
|  |  | 1 | Participants / Incident cases | 34,774 / 1596 | 11,150 / 586 | 11,152 / 450 |  |
|  |  |  | HR (95% CI) | 1 | 1.08 (0.98 to 1.20) | 1.09 (0.94 to 1.23) | 0.088 |
|  |  | 2 | Participants / Incident cases | 66,775 / 2033 | 17,107 / 713 | 17,123 / 473 |  |
|  |  |  | HR (95% CI) | 1 | 1.11 (1.01 to 1.21) | 1.16 (1.03 to 1.30) | 0.004 |
|  |  | 3 | Participants / Incident cases | 67,662 / 2096 | 17,601 / 766 | 17,602 / 496 |  |
|  |  |  | HR (95% CI) | 1 | 1.12 (1.02 to 1.22) | 1.13 (1.01 to 1.26) | 0.009 |
|  |  | 4 | Participants / Incident cases | 67,662 / 2096 | 17,601 / 766 | 17,602 / 496 |  |
|  |  |  | HR (95% CI) | 1 | 1.12 (1.03 to 1.22) | 1.13 (1.01 to 1.26) | 0.008 |
|  |  | 5 | Participants / Incident cases | 67,662 / 2096 | 17,601 / 766 | 17,602 / 496 |  |
|  |  |  | HR (95% CI) | 1 | 1.12 (1.03 to 1.22) | 1.13 (1.02 to 1.27) | 0.006 |
|  |  | 6 | Participants / Incident cases | 67,662 / 2096 | 17,601 / 766 | 17,602 / 496 |  |
|  |  |  | HR (95% CI) | 1 | 1.12 (1.03 to 1.22) | 1.13 (1.01 to 1.26) | 0.009 |
|  |  | 7 | Participants / Incident cases | 67,662 / 2096 | 17,601 / 766 | 17,602 / 496 |  |
|  |  |  | HR (95% CI) | 1 | 1.12 (1.03 to 1.22) | 1.13 (1.02 to 1.27) | 0.006 |
|  |  | 8 | Participants / Incident cases | 86,542 / 2901 | 17,574 / 798 | 17,562 / 519 |  |
|  |  |  | HR (95% CI) | 1 | 1.11 (1.02 to 1.20) | 1.12 (1.00 to 1.24) | 0.010 |
|  |  | 9 | Participants / Incident cases | 67,662 / 2096 | 17,601 / 766 | 17,602 / 496 |  |
|  |  |  | HR (95% CI) | 1 | 1.09 (1.00 to 1.19) | 1.16 (1.05 to 1.29) | 0.003 |
|  |  | 10 | Participants / Incident cases | 67,662 / 2096 | 17,601 / 766 | 17,602 / 496 |  |
|  |  |  | HR (95% CI) | 1 | 1.12 (1.02 to 1.22) | 1.13 (1.01 to 1.27) | 0.008 |
|  |  | 11 | Participants / Incident cases | 60,698 / 1574 | 16,341 / 577 | 16,345 / 376 |  |
|  |  |  | HR (95% CI) | 1 | 1.12 (1.01 to 1.24) | 1.11 (0.98 to 1.26) | 0.037 |
|  |  | 12 | Participants / Incident cases | 55,992 / 1825 | 14,469 / 653 | 14,471 / 430 |  |
|  |  |  | HR (95% CI) | 1 | 1.12 (1.02 to 1.23) | 1.12 (0.99 to 1.26) | 0.017 |
|  | Sucralose | Main | Participants / Incident cases | 88,867 / 2883 | 7005 / 288 | 6993 / 187 |  |
|  |  |  | HR (95% CI) | 1 | 1.03 (0.91 to 1.17) | 0.96 (0.82 to 1.12) | 0.823 |
|  |  | 1 | Participants / Incident cases | 46,998 / 2201 | 5040 / 225 | 5038 / 206 |  |
|  |  |  | HR (95% CI) | 1 | 1.01 (0.88 to 1.16) | 1.05 (0.90 to 1.22) | 0.543 |
|  |  | 2 | Participants / Incident cases | 87,298 / 2767 | 6837 / 272 | 6,870 / 180 |  |
|  |  |  | HR (95% CI) | 1 | 1.02 (0.89 to 1.15) | 0.97 (0.83 to 1.14) | 0.847 |
|  |  | 3 | Participants / Incident cases | 88,867 / 2883 | 7005 / 288 | 6993 / 187 |  |
|  |  |  | HR (95% CI) | 1 | 1.03 (0.91 to 1.17) | 0.96 (0.82 to 1.12) | 0.820 |
|  |  | 4 | Participants / Incident cases | 88,867 / 2883 | 7005 / 288 | 6993 / 187 |  |
|  |  |  | HR (95% CI) | 1 | 1.03 (0.91 to 1.17) | 0.95 (0.82 to 1.11) | 0.782 |
|  |  | 5 | Participants / Incident cases | 88,867 / 2883 | 7005 / 288 | 6993 / 187 |  |
|  |  |  | HR (95% CI) | 1 | 1.04 (0.92 to 1.17) | 0.96 (0.83 to 1.12) | 0.885 |
|  |  | 6 | Participants / Incident cases | 88,867 / 2883 | 7005 / 288 | 6993 / 187 |  |
|  |  |  | HR (95% CI) | 1 | 1.03 (0.91 to 1.17) | 0.95 (0.82 to 1.11) | 0.763 |
|  |  | 7 | Participants / Incident cases | 88,867 / 2883 | 7005 / 288 | 6993 / 187 |  |
|  |  |  | HR (95% CI) | 1 | 1.04 (0.91 to 1.17) | 0.96 (0.82 to 1.12) | 0.838 |
|  |  | 8 | Participants / Incident cases | 107,743 / 3713 | 6971 / 306 | 6964 / 199 |  |
|  |  |  | HR (95% CI) | 1 | 1.03 (0.92 to 1.16) | 0.98 (0.84 to 1.13) | 0.985 |
|  |  | 9 | Participants / Incident cases | 88,867 / 2883 | 7005 / 288 | 6993 / 187 |  |
|  |  |  | HR (95% CI) | 1 | 1.07 (0.97 to 1.18) | 1.04 (0.92 to 1.18) | 0.280 |
|  |  | 10 | Participants / Incident cases | 88,867 / 2883 | 7005 / 288 | 6993 / 187 |  |
|  |  |  | HR (95% CI) | 1 | 1.03 (0.91 to 1.17) | 0.96 (0.82 to 1.12) | 0.832 |
|  |  | 11 | Participants / Incident cases | 80,307 / 2170 | 6531 / 219 | 6546 / 138 |  |
|  |  |  | HR (95% CI) | 1 | 1.04 (0.90 to 1.20) | 0.90 (0.76 to 1.08) | 0.481 |
|  |  | 12 | Participants / Incident cases | 73,288 / 2494 | 5819 / 252 | 5825 / 162 |  |
|  |  |  | HR (95% CI) | 1 | 1.06 (0.92 to 1.20) | 0.98 (0.83 to 1.15) | 0.9039 |
| Breast cancer | Total artificial sweeteners | Main | Participants / Incident cases | 49,349 / 556 | 15,681 / 229 | 15,681 / 194 |  |
|  |  |  | HR (95% CI) | 1 | 1.11 (0.95 to 1.30) | 1.16 (0.97 to 1.38) | 0.064 |
|  |  | 1 | Participants / Incident cases | 24,668 / 421 | 9903 / 174 | 9899 / 174 |  |
|  |  |  | HR (95% CI) | 1 | 1.09 (0.91 to 1.30) | 1.13 (0.94 to 1.36) | 0.170 |
|  |  | 2 | Participants / Incident cases | 48,909 / 547 | 15,377 / 223 | 15,378 / 184 |  |
|  |  |  | HR (95% CI) | 1 | 1.12 (0.95 to 1.31) | 1.15 (0.97 to 1.37) | 0.078 |
|  |  | 3 | Participants / Incident cases | 49,349 / 556 | 15,681 / 229 | 15,681 / 194 |  |
|  |  |  | HR (95% CI) | 1 | 1.11 (0.95 to 1.30) | 1.16 (0.97 to 1.36) | 0.079 |
|  |  | 4 | Participants / Incident cases | 49,349 / 556 | 15,681 / 229 | 15,681 / 194 |  |
|  |  |  | HR (95% CI) | 1 | 1.11 (0.95 to 1.30) | 1.16 (0.98 to 1.38) | 0.065 |
|  |  | 5 | Participants / Incident cases | 49,349 / 556 | 15,681 / 229 | 15,681 / 194 |  |
|  |  |  | HR (95% CI) | 1 | 1.12 (0.95 to 1.31) | 1.16 (0.98 to 1.38) | 0.060 |
|  |  | 6 | Participants / Incident cases | 49,349 / 556 | 15,681 / 229 | 15,681 / 194 |  |
|  |  |  | HR (95% CI) | 1 | 1.12 (0.96 to 1.31) | 1.18 (0.99 to 1.42) | 0.042 |
|  |  | 7 | Participants / Incident cases | 49,349 / 556 | 15,681 / 229 | 15,681 / 194 |  |
|  |  |  | HR (95% CI) | 1 | 1.12 (0.96 to 1.31) | 1.18 (1.00 to 1.41) | 0.038 |
|  |  | 8 | Participants / Incident cases | 63,496 / 791 | 15,613 / 241 | 15,613 / 202 |  |
|  |  |  | HR (95% CI) | 1 | 1.05 (0.90 to 1.21) | 1.12 (0.95 to 1.32) | 0.156 |
|  |  | 9 | Participants / Incident cases | 49,349 / 556 | 15,681 / 229 | 15,681 / 194 |  |
|  |  |  | HR (95% CI) | 1 | 1.16 (1.00 to 1.36) | 1.22 (1.03 to 1.45) | 0.013 |
|  |  | 10 | Participants / Incident cases | 49,349 / 556 | 15,681 / 229 | 15,681 / 194 |  |
|  |  |  | HR (95% CI) | 1 | 1.10 (0.94 to 1.29) | 1.15 (0.97 to 1.37) | 0.074 |
|  |  | 11 | Participants / Incident cases | 49,196 / 394 | 15,633 / 181 | 15,634 / 146 |  |
|  |  |  | HR (95% CI) | 1 | 1.20 (1.00 to 1.43) | 1.21 (0.99 to 1.48) | 0.030 |
|  |  | 12 | Participants / Incident cases | 40,784 / 499 | 12,864 / 203 | 12,865 / 170 |  |
|  |  |  | HR (95% CI) | 1 | 1.12 (0.94 to 1.32) | 1.13 (0.94 to 1.35) | 0.139 |
|  | Aspartame | Main | Participants / Incident cases | 56,712 / 647 | 11,999 / 176 | 12,000 / 156 |  |
|  |  |  | HR (95% CI) | 1 | 1.09 (0.92 to 1.29) | 1.22 (1.01 to 1.48) | 0.036 |
|  |  | 1 | Participants / Incident cases | 29,560 / 503 | 7455 / 135 | 7455 / 131 |  |
|  |  |  | HR (95% CI) | 1 | 1.07 (0.88 to 1.30) | 1.07 (0.87 to 1.32) | 0.456 |
|  |  | 2 | Participants / Incident cases | 56,195 / 457 | 11,718 / 170 | 11,751 / 147 |  |
|  |  |  | HR (95% CI) | 1 | 1.09 (0.91 to 1.29) | 1.21 (0.99 to 1.46) | 0.052 |
|  |  | 3 | Participants / Incident cases | 56,712 / 647 | 11,999 / 176 | 12,000 / 156 |  |
|  |  |  | HR (95% CI) | 1 | 1.08 (0.91 to 1.29) | 1.20 (1.00 to 1.46) | 0.047 |
|  |  | 4 | Participants / Incident cases | 56,712 / 647 | 11,999 / 176 | 12,000 / 156 |  |
|  |  |  | HR (95% CI) | 1 | 1.09 (0.92 to 1.29) | 1.22 (1.01 to 1.48) | 0.036 |
|  |  | 5 | Participants / Incident cases | 56,712 / 647 | 11,999 / 176 | 12,000 / 156 |  |
|  |  |  | HR (95% CI) | 1 | 1.09 (0.92 to 1.30) | 1.22 (1.01 to 1.48) | 0.036 |
|  |  | 6 | Participants / Incident cases | 56,712 / 647 | 11,999 / 176 | 12,000 / 156 |  |
|  |  |  | HR (95% CI) | 1 | 1.10 (0.92 to 1.30) | 1.24 (1.02 to 1.50) | 0.027 |
|  |  | 7 | Participants / Incident cases | 56,712 / 647 | 11,999 / 176 | 12,000 / 156 |  |
|  |  |  | HR (95% CI) | 1 | 1.10 (0.93 to 1.30) | 1.25 (1.03 to 1.51) | 0.021 |
|  |  | 8 | Participants / Incident cases | 70,738 / 893 | 11,992 / 178 | 11,992 / 163 |  |
|  |  |  | HR (95% CI) | 1 | 0.99 (0.84 to 1.17) | 1.20 (1.00 to 1.43) | 0.107 |
|  |  | 9 | Participants / Incident cases | 56,712 / 647 | 11,999 / 176 | 12,000 / 156 |  |
|  |  |  | HR (95% CI) | 1 | 1.05 (0.89 to 1.23) | 1.18 (0.97 to 1.42) | 0.106 |
|  |  | 10 | Participants / Incident cases | 56,712 / 647 | 11,999 / 176 | 12,000 / 156 |  |
|  |  |  | HR (95% CI) | 1 | 1.08 (0.91 to 1.28) | 1.21 (1.00 to 1.47) | 0.050 |
|  |  | 11 | Participants / Incident cases | 56,537 / 463 | 11,962 / 157 | 11,964 / 101 |  |
|  |  |  | HR (95% CI) | 1 | 1.16 (0.96 to 1.41) | 1.28 (1.03 to 1.60) | 0.016 |
|  |  | 12 | Participants / Incident cases | 46,906 / 584 | 9803 / 155 | 9804 / 133 |  |
|  |  |  | HR (95% CI) | 1 | 1.09 (0.91 to 1.30) | 1.13 (0.92 to 1.39) | 0.182 |
|  | Acesulfame-K | Main | Participants / Incident cases | 51,554 / 581 | 14,578 / 232 | 14,579 / 166 |  |
|  |  |  | HR (95% CI) | 1 | 1.11 (0.95 to 1.30) | 1.17 (0.96 to 1.43) | 0.086 |
|  |  | 1 | Participants / Incident cases | 26,136 / 444 | 9166 / 174 | 9168 / 151 |  |
|  |  |  | HR (95% CI) | 1 | 1.05 (0.87 to 1.26) | 1.13 (0.92 to 1.40) | 0.250 |
|  |  | 2 | Participants / Incident cases | 51,089 / 571 | 14,282 / 221 | 14,293 / 162 |  |
|  |  |  | HR (95% CI) | 1 | 1.10 (0.93 to 1.29) | 1.20 (0.98 to 1.47) | 0.058 |
|  |  | 3 | Participants / Incident cases | 51,554 / 581 | 14,578 / 232 | 14,579 / 166 |  |
|  |  |  | HR (95% CI) | 1 | 1.11 (0.95 to 1.30) | 1.16 (0.95 to 1.42) | 0.095 |
|  |  | 4 | Participants / Incident cases | 51,554 / 581 | 14,578 / 232 | 14,579 / 166 |  |
|  |  |  | HR (95% CI) | 1 | 1.11 (0.95 to 1.30) | 1.17 (0.96 to 1.43) | 0.088 |
|  |  | 5 | Participants / Incident cases | 51,554 / 581 | 14,578 / 232 | 14,579 / 166 |  |
|  |  |  | HR (95% CI) | 1 | 1.11 (0.95 to 1.31) | 1.17 (0.96 to 1.43) | 0.080 |
|  |  | 6 | Participants / Incident cases | 51,554 / 581 | 14,578 / 232 | 14,579 / 166 |  |
|  |  |  | HR (95% CI) | 1 | 1.11 (0.95 to 1.31) | 1.19 (0.97 to 1.46) | 0.063 |
|  |  | 7 | Participants / Incident cases | 51,554 / 581 | 14,578 / 232 | 14,579 / 166 |  |
|  |  |  | HR (95% CI) | 1 | 1.12 (0.95 to 1.31) | 1.19 (0.97 to 1.45) | 0.059 |
|  |  | 8 | Participants / Incident cases | 65,619 / 819 | 14,555 / 245 | 14,548 / 170 |  |
|  |  |  | HR (95% CI) | 1 | 1.06 (0.91 to 1.23) | 1.11 (0.92 to 1.34) | 0.255 |
|  |  | 9 | Participants / Incident cases | 51,554 / 581 | 14,578 / 232 | 14,579 / 166 |  |
|  |  |  | HR (95% CI) | 1 | 1.09 (0.94 to 1.28) | 1.28 (1.05 to 1.55) | 0.014 |
|  |  | 10 | Participants / Incident cases | 51,554 / 581 | 14,578 / 232 | 14,579 / 166 |  |
|  |  |  | HR (95% CI) | 1 | 1.10 (0.94 to 1.29) | 1.16 (0.96 to 1.42) | 0.092 |
|  |  | 11 | Participants / Incident cases | 51,395 / 413 | 14,547 / 175 | 14,521 / 133 |  |
|  |  |  | HR (95% CI) | 1 | 1.16 (0.97 to 1.40) | 1.31 (1.04 to 1.65) | 0.014 |
|  |  | 12 | Participants / Incident cases | 42,597 / 524 | 11,957 / 205 | 11,959 / 143 |  |
|  |  |  | HR (95% CI) | 1 | 1.10 (0.93 to 1.31) | 1.10 (0.89 to 1.36) | 0.278 |
|  | Sucralose | Main | Participants / Incident cases | 69,189 / 826 | 5772 / 93 | 5750 / 60 |  |
|  |  |  | HR (95% CI) | 1 | 1.04 (0.84 to 1.30) | 0.93 (0.71 to 1.22) | 0.786 |
|  |  | 1 | Participants / Incident cases | 36,204 / 629 | 4133 / 72 | 4233 / 68 |  |
|  |  |  | HR (95% CI) | 1 | 1.02 (0.79 to 1.30) | 1.08 (0.83 to 1.40) | 0.585 |
|  |  | 2 | Participants / Incident cases | 68,337 / 804 | 5654 / 91 | 5673 / 59 |  |
|  |  |  | HR (95% CI) | 1 | 1.05 (0.84 to 1.31) | 0.96 (0.73 to 1.26) | 0.9602 |
|  |  | 3 | Participants / Incident cases | 69,189 / 826 | 5772 / 93 | 5750 / 60 |  |
|  |  |  | HR (95% CI) | 1 | 1.04 (0.84 to 1.30) | 0.93 (0.71 to 1.22) | 0.785 |
|  |  | 4 | Participants / Incident cases | 69,189 / 826 | 5772 / 93 | 5750 / 60 |  |
|  |  |  | HR (95% CI) | 1 | 1.04 (0.84 to 1.30) | 0.93 (0.71 to 1.22) | 0.774 |
|  |  | 5 | Participants / Incident cases | 69,189 / 826 | 5772 / 93 | 5750 / 60 |  |
|  |  |  | HR (95% CI) | 1 | 1.05 (0.84 to 1.30) | 0.94 (0.71 to 1.23) | 0.828 |
|  |  | 6 | Participants / Incident cases | 69,189 / 826 | 5772 / 93 | 5750 / 60 |  |
|  |  |  | HR (95% CI) | 1 | 1.04 (0.84 to 1.30) | 0.94 (0.71 to 1.23) | 0.822 |
|  |  | 7 | Participants / Incident cases | 69,189 / 826 | 5772 / 93 | 5750 / 60 |  |
|  |  |  | HR (95% CI) | 1 | 1.05 (0.84 to 1.30) | 0.94 (0.71 to 1.23) | 0.828 |
|  |  | 8 | Participants / Incident cases | 83,240 / 1067 | 5741 / 103 | 5741 / 64 |  |
|  |  |  | HR (95% CI) | 1 | 1.03 (0.84 to 1.27) | 0.95 (0.73 to 1.24) | 0.873 |
|  |  | 9 | Participants / Incident cases | 69,189 / 826 | 5772 / 93 | 5750 / 60 |  |
|  |  |  | HR (95% CI) | 1 | 1.27 (1.06 to 1.51) | 1.10 (0.88 to 1.38) | 0.077 |
|  |  | 10 | Participants / Incident cases | 69,189 / 826 | 5772 / 93 | 5750 / 60 |  |
|  |  |  | HR (95% CI) | 1 | 1.04 (0.83 to 1.29) | 0.94 (0.72 to 1.24) | 0.815 |
|  |  | 11 | Participants / Incident cases | 68,971 / 599 | 5651 / 69 | 5841 / 53 |  |
|  |  |  | HR (95% CI) | 1 | 1.08 (0.84 to 1.38) | 1.04 (0.77 to 1.40) | 0.653 |
|  |  | 12 | Participants / Incident cases | 56,916 / 733 | 4798 / 86 | 4799 / 53 |  |
|  |  |  | HR (95% CI) | 1 | 1.10 (0.88 to 1.38) | 0.93 (0.70 to 1.25) | 0.980 |
| Prostate cancer | Total artificial sweeteners | Main | Participants / Incident cases | 15,543 / 282 | 3305 / 63 | 3306 / 58 |  |
|  |  |  | HR (95% CI) | 1 | 0.92 (0.70 to 1.22) | 1.26 (0.94 to 1.68) | 0.274 |
|  |  | 1 | Participants / Incident cases | 8246 / 239 | 2178 / 49 | 2182 / 59 |  |
|  |  |  | HR (95% CI) | 1 | 0.87 (0.64 to 1.18) | 1.20 (0.89 to 1.62) | 0.430 |
|  |  | 2 | Participants / Incident cases | 15,146 / 270 | 3098 / 60 | 3097 / 50 |  |
|  |  |  | HR (95% CI) | 1 | 0.95 (0.71 to 1.26) | 1.25 (0.92 to 1.70) | 0.298 |
|  |  | 3 | Participants / Incident cases | 15,543 / 282 | 3305 / 63 | 3306 / 58 |  |
|  |  |  | HR (95% CI) | 1 | 0.92 (0.70 to 1.22) | 1.28 (0.96 to 1.72) | 0.220 |
|  |  | 4 | Participants / Incident cases | 15,543 / 282 | 3305 / 63 | 3306 / 58 |  |
|  |  |  | HR (95% CI) | 1 | 0.92 (0.70 to 1.21) | 1.24 (0.93 to 1.67) | 0.308 |
|  |  | 5 | Participants / Incident cases | 15,543 / 282 | 3305 / 63 | 3306 / 58 |  |
|  |  |  | HR (95% CI) | 1 | 0.92 (0.70 to 1.21) | 1.25 (0.93 to 1.67) | 0.302 |
|  |  | 6 | Participants / Incident cases | 15,543 / 282 | 3305 / 63 | 3306 / 58 |  |
|  |  |  | HR (95% CI) | 1 | 0.92 (0.70 to 1.21) | 1.25 (0.93 to 1.68) | 0.310 |
|  |  | 7 | Participants / Incident cases | 15,543 / 282 | 3305 / 63 | 3306 / 58 |  |
|  |  |  | HR (95% CI) | 1 | 0.92 (0.70 to 1.22) | 1.26 (0.94 to 1.60) | 0.274 |
|  |  | 8 | Participants / Incident cases | 20,386 / 379 | 3285 / 69 | 3285 / 62 |  |
|  |  |  | HR (95% CI) | 1 | 0.94 (0.72 to 1.21) | 1.26 (0.95 to 1.66) | 0.233 |
|  |  | 9 | Participants / Incident cases | 15,543 / 282 | 3305 / 63 | 3306 / 58 |  |
|  |  |  | HR (95% CI) | 1 | 0.97 (0.77 to 1.23) | 1.12 (0.84 to 1.49) | 0.593 |
|  |  | 10 | Participants / Incident cases | 15,543 / 282 | 3305 / 63 | 3306 / 58 |  |
|  |  |  | HR (95% CI) | 1 | 0.90 (0.68 to 1.19) | 1.23 (0.92 to 1.65) | 0.379 |
|  |  | 11 | Participants / Incident cases | 14,129 / 206 | 3054 / 51 | 3053 / 40 |  |
|  |  |  | HR (95% CI) | 1 | 1.05 (0.77 to 1.43) | 1.16 (0.82 to 1.65) | 0.404 |
|  |  | 12 | Participants / Incident cases | 12,929 / 262 | 2745 / 56 | 2745 / 56 |  |
|  |  |  | HR (95% CI) | 1 | 0.89 (0.66 to 1.19) | 1.23 (0.91 to 1.66) | 0.376 |
|  | Aspartame | Main | Participants / Incident cases | 17,457 / 310 | 2346 / 49 | 2351 / 44 |  |
|  |  |  | HR (95% CI) | 1 | 0.95 (0.70 to 1.30) | 1.28 (0.91 to 1.79) | 0.280 |
|  |  | 1 | Participants / Incident cases | 9542 / 265 | 1532 / 39 | 1532 / 43 |  |
|  |  |  | HR (95% CI) | 1 | 0.93 (0.66 to 1.31) | 1.19 (0.84 to 1.69) | 0.477 |
|  |  | 2 | Participants / Incident cases | 17,008 / 297 | 2,175 / 46 | 2,158 / 37 |  |
|  |  |  | HR (95% CI) | 1 | 0.97 (0.71 to 1.33) | 1.24 (0.87 to 1.77) | 0.367 |
|  |  | 3 | Participants / Incident cases | 17,457 / 310 | 2346 / 49 | 2351 / 44 |  |
|  |  |  | HR (95% CI) | 1 | 0.97 (0.71 to 1.31) | 1.31 (0.94 to 1.83) | 0.209 |
|  |  | 4 | Participants / Incident cases | 17,457 / 310 | 2346 / 49 | 2351 / 44 |  |
|  |  |  | HR (95% CI) | 1 | 0.95 (0.70 to 1.29) | 1.27 (0.91 to 1.78) | 0.300 |
|  |  | 5 | Participants / Incident cases | 17,457 / 310 | 2346 / 49 | 2351 / 44 |  |
|  |  |  | HR (95% CI) | 1 | 0.95 (0.70 to 1.29) | 1.27 (0.91 to 1.78) | 0.301 |
|  |  | 6 | Participants / Incident cases | 17,457 / 310 | 2346 / 49 | 2351 / 44 |  |
|  |  |  | HR (95% CI) | 1 | 0.95 (0.70 to 1.29) | 1.27 (0.90 to 1.78) | 0.309 |
|  |  | 7 | Participants / Incident cases | 17,457 / 310 | 2346 / 49 | 2351 / 44 |  |
|  |  |  | HR (95% CI) | 1 | 0.96 (0.70 to 1.30) | 1.28 (0.91 to 1.79) | 0.280 |
|  |  | 8 | Participants / Incident cases | 22,259 / 411 | 2348 / 52 | 2349 / 47 |  |
|  |  |  | HR (95% CI) | 1 | 0.94 (0.70 to 1.27) | 1.26 (0.91 to 1.73) | 0.302 |
|  |  | 9 | Participants / Incident cases | 17,457 / 310 | 2346 / 49 | 2351 / 44 |  |
|  |  |  | HR (95% CI) | 1 | 1.12 (0.87 to 1.45) | 1.21 (0.87 to 1.71) | 0.194 |
|  |  | 10 | Participants / Incident cases | 17,457 / 310 | 2346 / 49 | 2351 / 44 |  |
|  |  |  | HR (95% CI) | 1 | 0.94 (0.69 to 1.27) | 1.26 (0.89 to 1.77) | 0.358 |
|  |  | 11 | Participants / Incident cases | 15,871 / 228 | 2182 / 38 | 2183 / 31 |  |
|  |  |  | HR (95% CI) | 1 | 1.03 (0.73 to 1.46) | 1.20 (0.80 to 1.79) | 0.408 |
|  |  | 12 | Participants / Incident cases | 14,500 / 286 | 1969 / 45 | 1950 / 43 |  |
|  |  |  | HR (95% CI) | 1 | 0.95 (0.69 to 1.31) | 1.27 (0.90 to 1.79) | 0.294 |
|  | Acesulfame-K | Main | Participants / Incident cases | 16,108 / 288 | 3023 / 76 | 3023 / 39 |  |
|  |  |  | HR (95% CI) | 1 | 1.06 (0.81 to 1.39) | 1.18 (0.82 to 1.71) | 0.365 |
|  |  | 1 | Participants / Incident cases | 8638 / 245 | 1984 / 66 | 1984 / 36 |  |
|  |  |  | HR (95% CI) | 1 | 1.09 (0.82 to 1.45) | 0.98 (0.67 to 1.43) | 0.891 |
|  |  | 2 | Participants / Incident cases | 15,696 / 275 | 2825 / 69 | 2830 / 36 |  |
|  |  |  | HR (95% CI) | 1 | 1.05 (0.79 to 1.38) | 1.25 (0.85 to 1.83) | 0.297 |
|  |  | 3 | Participants / Incident cases | 16,108 / 288 | 3023 / 76 | 3023 / 39 |  |
|  |  |  | HR (95% CI) | 1 | 1.06 (0.81 to 1.39) | 1.20 (0.83 to 1.73) | 0.334 |
|  |  | 4 | Participants / Incident cases | 16,108 / 288 | 3023 / 76 | 3023 / 39 |  |
|  |  |  | HR (95% CI) | 1 | 1.06 (0.81 to 1.38) | 1.17 (0.81 to 1.69) | 0.403 |
|  |  | 5 | Participants / Incident cases | 16,108 / 288 | 3023 / 76 | 3023 / 39 |  |
|  |  |  | HR (95% CI) | 1 | 1.06 (0.81 to 1.38) | 1.17 (0.81 to 1.68) | 0.401 |
|  |  | 6 | Participants / Incident cases | 16,108 / 288 | 3023 / 76 | 3023 / 39 |  |
|  |  |  | HR (95% CI) | 1 | 1.06 (0.81 to 1.38) | 1.17 (0.80 to 1.70) | 0.406 |
|  |  | 7 | Participants / Incident cases | 16,108 / 288 | 3023 / 76 | 3023 / 39 |  |
|  |  |  | HR (95% CI) | 1 | 1.06 (0.81 to 1.39) | 1.18 (0.82 to 1.71) | 0.366 |
|  |  | 8 | Participants / Incident cases | 20,923 / 386 | 3019 / 84 | 3014 / 40 |  |
|  |  |  | HR (95% CI) | 1 | 1.09 (0.85 to 1.40) | 1.12 (0.79 to 1.60) | 0.411 |
|  |  | 9 | Participants / Incident cases | 16,108 / 288 | 3023 / 76 | 3023 / 39 |  |
|  |  |  | HR (95% CI) | 1 | 1.12 (0.89 to 1.42) | 1.04 (0.73 to 1.47) | 0.554 |
|  |  | 10 | Participants / Incident cases | 16,108 / 288 | 3023 / 76 | 3023 / 39 |  |
|  |  |  | HR (95% CI) | 1 | 1.04 (0.80 to 1.34) | 1.14 (0.79 to 1.65) | 0.489 |
|  |  | 11 | Participants / Incident cases | 14,632 / 211 | 2800 / 56 | 2804 / 30 |  |
|  |  |  | HR (95% CI) | 1 | 1.11 (0.81 to 1.52) | 1.25 (0.82 to 1.91) | 0.268 |
|  |  | 12 | Participants / Incident cases | 13,395 / 266 | 2512 / 73 | 2512 / 35 |  |
|  |  |  | HR (95% CI) | 1 | 1.10 (0.84 to 1.45) | 1.04 (0.71 to 1.52) | 0.655 |
|  | Sucralose | Main | Participants / Incident cases | 19,678 / 365 | 1233 / 25 | 1243 / 13 |  |
|  |  |  | HR (95% CI) | 1 | 0.86 (0.57 to 1.30) | 1.01 (0.57 to 1.77) | 0.699 |
|  |  | 1 | Participants / Incident cases | 10,794 / 312 | 907 / 20 | 905 / 15 |  |
|  |  |  | HR (95% CI) | 1 | 0.81 (0.51 to 1.27) | 0.97 (0.57 to 1.66) | 0.592 |
|  |  | 2 | Participants / Incident cases | 18,961 / 343 | 1183 / 25 | 1197 / 12 |  |
|  |  |  | HR (95% CI) | 1 | 0.90 (0.60 to 1.37) | 1.02 (0.56 to 1.84) | 0.8192 |
|  |  | 3 | Participants / Incident cases | 19,678 / 365 | 1233 / 25 | 1243 / 13 |  |
|  |  |  | HR (95% CI) | 1 | 0.85 (0.57 to 1.29) | 0.98 (0.56 to 1.73) | 0.636 |
|  |  | 4 | Participants / Incident cases | 19,678 / 365 | 1233 / 25 | 1243 / 13 |  |
|  |  |  | HR (95% CI) | 1 | 0.85 (0.57 to 1.29) | 0.98 (0.56 to 1.73) | 0.642 |
|  |  | 5 | Participants / Incident cases | 19,678 / 365 | 1233 / 25 | 1243 / 13 |  |
|  |  |  | HR (95% CI) | 1 | 0.86 (0.57 to 1.29) | 1.00 (0.57 to 1.76) | 0.687 |
|  |  | 6 | Participants / Incident cases | 19,678 / 365 | 1233 / 25 | 1243 / 13 |  |
|  |  |  | HR (95% CI) | 1 | 0.86 (0.57 to 1.29) | 0.99 (0.56 to 1.75) | 0.664 |
|  |  | 7 | Participants / Incident cases | 19,678 / 365 | 1233 / 25 | 1243 / 13 |  |
|  |  |  | HR (95% CI) | 1 | 0.86 (0.57 to 1.30) | 1.01 (0.57 to 1.77) | 0.700 |
|  |  | 8 | Participants / Incident cases | 24,503 / 468 | 1230 / 27 | 1223 / 15 |  |
|  |  |  | HR (95% CI) | 1 | 0.86 (0.58 to 1.28) | 1.06 (0.63 to 1.80) | 0.812 |
|  |  | 9 | Participants / Incident cases | 19,678 / 365 | 1233 / 25 | 1243 / 13 |  |
|  |  |  | HR (95% CI) | 1 | 0.97 (0.73 to 1.27) | 1.07 (0.72 to 1.59) | 0.904 |
|  |  | 10 | Participants / Incident cases | 19,678 / 365 | 1233 / 25 | 1243 / 13 |  |
|  |  |  | HR (95% CI) | 1 | 0.85 (0.56 to 1.27) | 0.98 (0.56 to 1.73) | 0.6246 |
|  |  | 11 | Participants / Incident cases | 17,936 / 268 | 1138 / 18 | 1162 / 11 |  |
|  |  |  | HR (95% CI) | 1 | 0.87 (0.54 to 1.46) | 1.16 (0.63 to 2.15) | 0.949 |
|  |  | 12 | Participants / Incident cases | 16,372 / 338 | 1021 / 23 | 1026 / 13 |  |
|  |  |  | HR (95% CI) | 1 | 0.83 (0.54 to 1.28) | 1.08 (0.61 to 1.89) | 0.793 |
| Obesity-related cancers | Total artificial sweeteners | Main | Participants / Incident cases | 64,892 / 1232 | 18,986 / 433 | 18,987 / 358 |  |
|  |  |  | HR (95% CI) | 1 | 1.08 (0.97 to 1.21) | 1.13 (1.00 to 1.28) | 0.036 |
|  |  | 1 | Participants / Incident cases | 32,914 / 942 | 12,081 / 327 | 12,081 / 322 |  |
|  |  |  | HR (95% CI) | 1 | 1.05 (0.92 to 1.19) | 1.08 (0.94 to 1.23) | 0.254 |
|  |  | 2 | Participants / Incident cases | 64,055 / 1195 | 18,475 / 416 | 18,475 / 327 |  |
|  |  |  | HR (95% CI) | 1 | 1.10 (0.98 to 1.23) | 1.12 (0.98 to 1.27) | 0.045 |
|  |  | 3 | Participants / Incident cases | 64,892 / 1232 | 18,986 / 433 | 18,987 / 358 |  |
|  |  |  | HR (95% CI) | 1 | 1.08 (0.97 to 1.21) | 1.13 (1.00 to 1.27) | 0.035 |
|  |  | 4 | Participants / Incident cases | 64,892 / 1232 | 18,986 / 433 | 18,987 / 358 |  |
|  |  |  | HR (95% CI) | 1 | 1.08 (0.97 to 1.21) | 1.13 (0.99 to 1.27) | 0.039 |
|  |  | 5 | Participants / Incident cases | 64,892 / 1232 | 18,986 / 433 | 18,987 / 358 |  |
|  |  |  | HR (95% CI) | 1 | 1.09 (0.97 to 1.22) | 1.13 (1.00 to 1.28) | 0.034 |
|  |  | 6 | Participants / Incident cases | 64,892 / 1232 | 18,986 / 433 | 18,987 / 358 |  |
|  |  |  | HR (95% CI) | 1 | 1.09 (0.97 to 1.22) | 1.13 (1.00 to 1.28) | 0.036 |
|  |  | 7 | Participants / Incident cases | 64,892 / 1232 | 18,986 / 433 | 18,987 / 358 |  |
|  |  |  | HR (95% CI) | 1 | 1.09 (0.97 to 1.22) | 1.14 (1.01 to 1.29) | 0.026 |
|  |  | 8 | Participants / Incident cases | 83,882 / 1740 | 18,898 / 456 | 18,898 / 377 |  |
|  |  |  | HR (95% CI) | 1 | 1.05 (0.95 to 1.17) | 1.11 (0.99 to 1.25) | 0.053 |
|  |  | 9 | Participants / Incident cases | 64,892 / 1232 | 18,986 / 433 | 18,987 / 358 |  |
|  |  |  | HR (95% CI) | 1 | 1.10 (0.98 to 1.22) | 1.13 (1.00 to 1.27) | 0.031 |
|  |  | 10 | Participants / Incident cases | 64,892 / 1232 | 18,986 / 433 | 18,987 / 358 |  |
|  |  |  | HR (95% CI) | 1 | 1.08 (0.96 to 1.20) | 1.12 (0.99 to 1.27) | 0.049 |
|  |  | 11 | Participants / Incident cases | 58,243 / 909 | 17,602 / 331 | 17,539 / 269 |  |
|  |  |  | HR (95% CI) | 1 | 1.10 (0.97 to 1.26) | 1.11 (0.96 to 1.28) | 0.098 |
|  |  | 12 | Participants / Incident cases | 53,713 / 1100 | 15,609 / 382 | 15,610 / 324 |  |
|  |  |  | HR (95% CI) | 1 | 1.09 (0.97 to 1.23) | 1.15 (1.01 to 1.31) | 0.020 |
|  | Aspartame | Main | Participants / Incident cases | 74,169 / 1401 | 14,345 / 337 | 14,351 / 285 |  |
|  |  |  | HR (95% CI) | 1 | 1.08 (0.96 to 1.22) | 1.15 (1.01 to 1.32) | 0.026 |
|  |  | 1 | Participants / Incident cases | 39,102 / 1092 | 8987 / 253 | 8987 / 246 |  |
|  |  |  | HR (95% CI) | 1 | 1.04 (0.91 to 1.20) | 1.05 (0.91 to 1.22) | 0.451 |
|  |  | 2 | Participants / Incident cases | 73,203 / 1360 | 13,893 / 318 | 13,909 / 260 |  |
|  |  |  | HR (95% CI) | 1 | 1.09 (0.96 to 1.23) | 1.16 (1.01 to 1.34) | 0.024 |
|  |  | 3 | Participants / Incident cases | 74,169 / 1401 | 14,345 / 337 | 14,351 / 285 |  |
|  |  |  | HR (95% CI) | 1 | 1.08 (0.96 to 1.22) | 1.15 (1.01 to 1.32) | 0.026 |
|  |  | 4 | Participants / Incident cases | 74,169 / 1401 | 14,345 / 337 | 14,351 / 285 |  |
|  |  |  | HR (95% CI) | 1 | 1.08 (0.96 to 1.22) | 1.15 (1.01 to 1.32) | 0.027 |
|  |  | 5 | Participants / Incident cases | 74,169 / 1401 | 14,345 / 337 | 14,351 / 285 |  |
|  |  |  | HR (95% CI) | 1 | 1.08 (0.96 to 1.22) | 1.15 (1.01 to 1.32) | 0.026 |
|  |  | 6 | Participants / Incident cases | 74,169 / 1401 | 14,345 / 337 | 14,351 / 285 |  |
|  |  |  | HR (95% CI) | 1 | 1.09 (0.96 to 1.23) | 1.16 (1.01 to 1.33) | 0.024 |
|  |  | 7 | Participants / Incident cases | 74,169 / 1401 | 14,345 / 337 | 14,351 / 285 |  |
|  |  |  | HR (95% CI) | 1 | 1.09 (0.96 to 1.23) | 1.17 (1.02 to 1.34) | 0.018 |
|  |  | 8 | Participants / Incident cases | 92,997 / 1930 | 14,340 / 344 | 14,341 / 299 |  |
|  |  |  | HR (95% CI) | 1 | 1.03 (0.91 to 1.15) | 1.14 (1.00 to 1.30) | 0.060 |
|  |  | 9 | Participants / Incident cases | 74,169 / 1401 | 14,345 / 337 | 14,351 / 285 |  |
|  |  |  | HR (95% CI) | 1 | 1.07 (0.96 to 1.20) | 1.13 (0.99 to 1.30) | 0.046 |
|  |  | 10 | Participants / Incident cases | 74,169 / 1401 | 14,345 / 337 | 14,351 / 285 |  |
|  |  |  | HR (95% CI) | 1 | 1.07 (0.95 to 1.21) | 1.15 (1.00 to 1.32) | 0.035 |
|  |  | 11 | Participants / Incident cases | 66,674 / 1034 | 13,355 / 259 | 13,355 / 216 |  |
|  |  |  | HR (95% CI) | 1 | 1.11 (0.97 to 1.28) | 1.14 (0.98 to 1.34) | 0.049 |
|  |  | 12 | Participants / Incident cases | 61,406 / 1254 | 11,772 / 298 | 11,754 / 254 |  |
|  |  |  | HR (95% CI) | 1 | 1.10 (0.96 to 1.25) | 1.17 (1.01 to 1.35) | 0.022 |
|  | Acesulfame-K | Main | Participants / Incident cases | 67,662 / 1275 | 17,601 / 457 | 17,602 / 291 |  |
|  |  |  | HR (95% CI) | 1 | 1.09 (0.97 to 1.22) | 1.13 (0.97 to 1.30) | 0.064 |
|  |  | 1 | Participants / Incident cases | 34,774 / 981 | 11,150 / 356 | 11,152 / 254 |  |
|  |  |  | HR (95% CI) | 1 | 1.06 (0.94 to 1.21) | 1.03 (0.88 to 1.21) | 0.519 |
|  |  | 2 | Participants / Incident cases | 66,775 / 1235 | 17,107 / 425 | 17,123 / 278 |  |
|  |  |  | HR (95% CI) | 1 | 1.08 (0.96 to 1.22) | 1.18 (1.02 to 1.37) | 0.021 |
|  |  | 3 | Participants / Incident cases | 67,662 / 1275 | 17,601 / 457 | 17,602 / 291 |  |
|  |  |  | HR (95% CI) | 1 | 1.09 (0.97 to 1.21) | 1.12 (0.97 to 1.30) | 0.066 |
|  |  | 4 | Participants / Incident cases | 67,662 / 1275 | 17,601 / 457 | 17,602 / 291 |  |
|  |  |  | HR (95% CI) | 1 | 1.09 (0.97 to 1.21) | 1.12 (0.97 to 1.30) | 0.070 |
|  |  | 5 | Participants / Incident cases | 67,662 / 1275 | 17,601 / 457 | 17,602 / 291 |  |
|  |  |  | HR (95% CI) | 1 | 1.09 (0.97 to 1.22) | 1.13 (0.98 to 1.30) | 0.061 |
|  |  | 6 | Participants / Incident cases | 67,662 / 1275 | 17,601 / 457 | 17,602 / 291 |  |
|  |  |  | HR (95% CI) | 1 | 1.09 (0.97 to 1.22) | 1.13 (0.98 to 1.31) | 0.060 |
|  |  | 7 | Participants / Incident cases | 67,662 / 1275 | 17,601 / 457 | 17,602 / 291 |  |
|  |  |  | HR (95% CI) | 1 | 1.09 (0.97 to 1.22) | 1.14 (0.98 to 1.31) | 0.050 |
|  |  | 8 | Participants / Incident cases | 86,542 / 1788 | 17,574 / 480 | 17,562 / 305 |  |
|  |  |  | HR (95% CI) | 1 | 1.07 (0.96 to 1.19) | 1.10 (0.96 to 1.27) | 0.101 |
|  |  | 9 | Participants / Incident cases | 67,662 / 1275 | 17,601 / 457 | 17,602 / 291 |  |
|  |  |  | HR (95% CI) | 1 | 1.06 (0.96 to 1.18) | 1.13 (0.99 to 1.30) | 0.065 |
|  |  | 10 | Participants / Incident cases | 67,662 / 1275 | 17,601 / 457 | 17,602 / 291 |  |
|  |  |  | HR (95% CI) | 1 | 1.08 (0.97 to 1.21) | 1.12 (0.96 to 1.29) | 0.087 |
|  |  | 11 | Participants / Incident cases | 60,698 / 941 | 16,341 / 337 | 16,345 / 231 |  |
|  |  |  | HR (95% CI) | 1 | 1.08 (0.95 to 1.23) | 1.18 (1.00 to 1.39) | 0.046 |
|  |  | 12 | Participants / Incident cases | 55,992 / 1140 | 14,469 / 408 | 14,471 / 258 |  |
|  |  |  | HR (95% CI) | 1 | 1.12 (1.00 to 1.26) | 1.12 (0.96 to 1.31) | 0.059 |
|  | Sucralose | Main | Participants / Incident cases | 88,867 / 1756 | 7005 / 167 | 6993 / 100 |  |
|  |  |  | HR (95% CI) | 1 | 0.98 (0.84 to 1.16) | 0.87 (0.71 to 1.07) | 0.230 |
|  |  | 1 | Participants / Incident cases | 46,998 / 1349 | 5040 / 130 | 5038 / 112 |  |
|  |  |  | HR (95% CI) | 1 | 0.95 (0.80 to 1.15) | 0.97 (0.80 to 1.19) | 0.666 |
|  |  | 2 | Participants / Incident cases | 87,298 / 1684 | 6837 / 158 | 6,870 / 96 |  |
|  |  |  | HR (95% CI) | 1 | 0.97 (0.82 to 1.14) | 0.89 (0.72 to 1.10) | 0.291 |
|  |  | 3 | Participants / Incident cases | 88,867 / 1756 | 7005 / 167 | 6993 / 100 |  |
|  |  |  | HR (95% CI) | 1 | 0.98 (0.84 to 1.15) | 0.87 (0.70 to 1.07) | 0.219 |
|  |  | 4 | Participants / Incident cases | 88,867 / 1756 | 7005 / 167 | 6993 / 100 |  |
|  |  |  | HR (95% CI) | 1 | 0.98 (0.83 to 1.15) | 0.86 (0.70 to 1.07) | 0.208 |
|  |  | 5 | Participants / Incident cases | 88,867 / 1756 | 7005 / 167 | 6993 / 100 |  |
|  |  |  | HR (95% CI) | 1 | 0.98 (0.84 to 1.16) | 0.87 (0.71 to 1.08) | 0.246 |
|  |  | 6 | Participants / Incident cases | 88,867 / 1756 | 7005 / 167 | 6993 / 100 |  |
|  |  |  | HR (95% CI) | 1 | 0.98 (0.84 to 1.15) | 0.87 (0.70 to 1.07) | 0.221 |
|  |  | 7 | Participants / Incident cases | 88,867 / 1756 | 7005 / 167 | 6993 / 100 |  |
|  |  |  | HR (95% CI) | 1 | 0.98 (0.84 to 1.16) | 0.87 (0.71 to 1.07) | 0.241 |
|  |  | 8 | Participants / Incident cases | 107,743 / 2284 | 6871 / 180 | 6964 / 109 |  |
|  |  |  | HR (95% CI) | 1 | 0.98 (0.84 to 1.15) | 0.90 (0.74 to 1.10) | 0.343 |
|  |  | 9 | Participants / Incident cases | 88,867 / 1756 | 7005 / 167 | 6993 / 100 |  |
|  |  |  | HR (95% CI) | 1 | 1.09 (0.96 to 1.24) | 0.99 (0.84 to 1.16) | 0.642 |
|  |  | 10 | Participants / Incident cases | 88,867 / 1756 | 7005 / 167 | 6993 / 100 |  |
|  |  |  | HR (95% CI) | 1 | 0.98 (0.83 to 1.15) | 0.87 (0.70 to 1.07) | 0.214 |
|  |  | 11 | Participants / Incident cases | 80,307 / 1306 | 6531 / 126 | 6546 / 77 |  |
|  |  |  | HR (95% CI) | 1 | 0.98 (0.82 to 1.18) | 0.86 (0.68 to 1.09) | 0.243 |
|  |  | 12 | Participants / Incident cases | 73,288 / 1563 | 5819 / 155 | 5825 / 88 |  |
|  |  |  | HR (95% CI) | 1 | 1.04 (0.88 to 1.23) | 0.88 (0.71 to 1.10) | 0.4581 |

^1^Median follow-up times for overall, breast, prostate and obesity-related cancers were, respectively, 7.8, 7.7, 8.8 and 7.7 y. Person-years were, respectively 708,076, 551,546, 156,993 and 708,392. Multivariable Cox proportional hazard models were adjusted for (= main model) age (time-scale), sex (except for breast and prostate), BMI (continuous, kg/m2), height (continuous, cm), percentage of weight gain during follow-up (continuous), physical activity (categorical International Physical Activity Questionnaire variable: high, moderate, low, missing value), smoking status (categorical: never, former, current smokers), number of smoked cigarettes in pack-years (continuous), educational level (categorical: less than high school degree, <2 y after high school degree, ≥2 y after high school degree), number of 24h dietary records (continuous), family history of cancer (categorical: yes, no), prevalent diabetes (categorical: yes, no), energy intake without alcohol (continuous variable : kcal/d), daily intakes (continuous, g/d) of: alcohol, sodium, saturated fatty acids, fibre, sugar, fruit and vegetable, whole-grain foods and dairy products. Breast cancer models were also adjusted for age at menarche (categorical: <12 y old, ≥12 y old), age at first child (categorical: no child, before 30 y, ≥30 y), number of biological children (continuous), baseline menopausal status (categorical: menopausal, non-menopausal), oral contraceptive use at baseline and during follow-up (categorical: yes, no), and hormonal treatment for menopause at baseline and during follow-up (categorical: yes, no).

In addition, all models were mutually adjusted for artificial sweetener intake other than the one studied.

^2^Sex specific cut-offs among consumers were 17.35 mg/d in men and 19.00 mg/d in women for total artificial sweeteners, 14,45 mg/d in men and 15.39 mg/d in women for aspartame, 5.11 mg/d in men and 5.51 mg/d in women for acesulfame-K and 3.46 mg/d in men and 3.43 mg/d in women for sucralose.

Model 1: main model, with restriction of the study population to participants with at least four 24h-dietary records during the first two years.

Model 2: main model, excluding participants with prevalent diabetes.

Model 3: main model, adjusted for “Healthy” and “Western” dietary patterns derived by Principal Component Analysis, not adjusted for fruit and vegetable, whole food and dairy products intakes.

Model 4: main model, additionally adjusted for consumption of sugary drinks (continuous, ml/d).

Model 5: main model, adjusted for added sugar intakes (continuous, g/d), not adjusted for sugar intakes.

Model 6: main model, additionally adjusted for the proportion of ultra-processed foods intake (continuous, % of ultra-processed in the diet).

Model 7: main model, additionally adjusted for weight-loss diet during the first two years of follow-up (categorical: yes, no).

Model 8: main model, not excluding under-energy reporters.

Model 9: main model, with artificial sweetener exposures coded as time-dependent variables.

Model 10: main model, using Fine and Gray subdistribution hazard model to deal with competing risks.

Model 11: main model, excluding the first two years of follow-up.

Model 12: main model, restricting the population study to non-smokers.

## References:

1. Black AE. Critical evaluation of energy intake using the Goldberg cut-off for energy intake:basal metabolic rate. A practical guide to its calculation, use and limitations. IntJObesRelat Metab Disord. 2000 Sep;24(0307-0565 (Print)):1119–30.

2. Black AE. The sensitivity and specificity of the Goldberg cut-off for EI:BMR for identifying diet reports of poor validity. European Journal of Clinical Nutrition. 2000 May;54(5):395–404.

3. Goldberg GR, Black AE, Jebb SA, Cole TJ, Murgatroyd PR, Coward WA, et al. Critical evaluation of energy intake data using fundamental principles of energy physiology: 1. Derivation of cut-off limits to identify under-recording. Eur J Clin Nutr. 1991 Dec;45(12):569–81.

4. Schofield WN. Predicting basal metabolic rate, new standards and review of previous work. HumNutr Clin Nutr. 1985;39 Suppl 1(0263-8290 (Print)):5–41.

5. Anses. Etude Individuelle Nationale des Consommations Alimentaires 3 (INCA 3). 2017.

6. Sterne JAC, White IR, Carlin JB, Spratt M, Royston P, Kenward MG, et al. Multiple imputation for missing data in epidemiological and clinical research: potential and pitfalls. BMJ. 2009 Jun 29;338:b2393.

7. Anses. Actualisation des repères du PNNS : établissement de recommandations d’apport en sucres. Maisons-Alfort: Anses; 2016 Dec p. 96.
