# Supplementary material for: Artificial sweeteners and cancer risk: Results from the NutriNet-Santé population-based cohort study
Source: PLoS Med. 2022 Mar 24;19(3):e1003950. doi: 10.1371/journal.pmed.1003950 (PMC8946744; doi:10.1371/journal.pmed.1003950)
Supplement: Attachment — Submitted filename: AUTHOR RESPONSE LETTER_PlosMed_R1_MT_CD_mt.docx [file pmed.1003950.s003.docx]

Author Response Letter

**Point-by-point Response**

We would like to sincerely thank the Editors and the Reviewers for granting us the opportunity to revise this paper. Their valuable comments helped us improve substantially the scientific quality and the readability of the paper. Kindly find below our point-by-point response.

The references to page/line numbers correspond to the new clean version of the manuscript.

We also went through the **Guidelines for revised papers** and made the requested edits throughout the manuscript.

**Comments from the academic editor:**

‘The dietary assessment method for this study is unique and strong - better than most other cohort studies. However, I have some concerns about potential residual confounding effects. The authors need to carefully address this issue in the revision’

We thank the Academic Editor for this overall positive comment.

Although residual confounding cannot be entirely ruled out (as in any epidemiological study), we have carefully adjusted the analyses for a wide range of potential confounders: socio-demographic factors (age, sex, educational level, physical activity, smoking), anthropometric factors (BMI, height, weight gain during follow-up), dietary factors (intakes of alcohol, sodium, saturated fatty acids, fibre, sugar, whole-grain foods and dairy products) and health-related factors (family history of cancer, diabetes, oral contraception, menopausal status, hormonal treatment). As requested, we have added in the abstract (p.2, l.39) the main dependent variables and mentioned residual confounding as a potential limitation.

We have also performed further adjusted models for dietary patterns, proportion of ultra-processed foods, and weight-loss diets and verified that results were similar when participants with diabetes were excluded (Supplementary Table 6).

Since smoking status is an important risk factors for some cancers we have now additionally tested a model restricting the population study to non-smokers, which provided similar results (Supplementary Table 6, model 12).

Some elements below provide additional information regarding potential residual confounding and how we have addressed this issue. In particular, as recommended by Reviewer #2 we have shown that the results were broadly very similar between a minimally adjusted model (only adjusted for age and sex) and the fully adjusted model (Supplementary Figure 2).

**Requests from the editors:**

Please revise your title according to PLOS Medicine's style. Your title must be nondeclarative and not a question. It should begin with main concept if possible. "Effect of" should be used only if causality can be inferred, i.e., for an RCT. Please place the study design ("A cohort study") in the subtitle (ie, after a colon).

The title has been changed to “Artificial sweeteners and cancer risk: results from the prospective NutriNet-Santé cohort” (p.1, l.4).

* Please structure your abstract using the PLOS Medicine headings (Background, Methods and Findings, Conclusions).

The abstract is now structured using the PLOS Medicine headings.

* Please combine the Methods and Findings sections into one section, “Methods and findings”.

Methods and findings have been combined into one section. (p.2, l.35)

Abstract Methods and Findings:

* Please include the study design and length of follow up.

Study design “French population-based prospective cohort NutriNet-Santé” and length of follow-up “median follow-up time=7.8y” have been added p.2, l.35.

* Please include the important dependent variables that are adjusted for in the analyses.

Main adjustment factors have been added to the abstract, p.2, l.39 “Associations between sweeteners and cancer incidence were assessed by Cox hazard models, notably adjusted for age, sex, education, physical activity, smoking, body mass index, height, weight-gain during follow-up, diabetes, family history of cancer, baseline intakes of energy, alcohol, sodium, saturated fatty acids, fibre, sugar, whole-grain foods and dairy products.”

* In the last sentence of the Abstract Methods and Findings section, please describe the main limitation(s) of the study's methodology.

The main limitations of the study’s methodology have been added at the end of the Methods and findings section, p.2, l.49: “Limitations of this study include potential selection bias, residual confounding, and reverse causality. However, several sensitivity analyses were performed to challenge these biases and provided very similar results, illustrating the robustness of the findings.”

At this stage, we ask that you include a short, non-technical Author Summary of your research to make findings accessible to a wide audience that includes both scientists and non-scientists. The Author Summary should immediately follow the Abstract in your revised manuscript. This text is subject to editorial change and should be distinct from the scientific abstract. Please see our author guidelines for more information: <https://journals.plos.org/plosmedicine/s/revising-your-manuscript#loc-author-summary>

An “Author Summary” section has been added according to the guidelines, p.3, l.59.

“**Author Summary – Why was this study done?**

- The food industry uses artificial sweeteners in a wide range of foods and beverages, as alternatives to added sugars for which deleterious effects on several chronic diseases are now well established.
- However, the safety of artificial sweeteners is questioned and their role in the aetiology of various diseases is debated.
- In particular, their carcinogenicity has been suggested by several experimental studies but robust epidemiological evidence is lacking.
- Previous observational studies have only investigated the associations between cancer risk and the consumption of artificially sweetened beverages used as a proxy.

**What did the researchers do and find?**

- In this large prospective cohort of 102,865 French adults, artificial sweeteners (especially aspartame and acesulfame-K) were associated with increased overall cancer risk.
- More specifically, aspartame intake was associated with increased breast and obesity-related cancer risks.

**What do these findings mean?**

- These results suggest that artificial sweeteners, used in thousands of food and beverage brands worldwide may represent a modifiable risk factor for cancer prevention.
- These findings provide novel information in the context of the ongoing re-evaluation of food additive sweeteners by the European Food Safety Authority and other health agencies globally.”

Citations should be in square brackets, and preceding punctuation.

References are now in the requested PLOS format.

Supplementary Figure 1 is central to the understanding of the paper. Please incorporate it into the main paper.

The Flowchart figure is now part of the main manuscript, and is referred to as Figure 1 throughout the text, and all Figures and Supplementary Figures have been renamed accordingly.

Please remove references to results tables from the methods (lines 177, 192, 205, 215, and 227).

References to tables was removed from the text where indicated.

Please ensure that the study is reported according to the STROBE guideline, and include the completed STROBE checklist as Supporting Information. Please add the following statement, or similar, to the Methods: "This study is reported as per the Strengthening the Reporting of Observational Studies in Epidemiology (STROBE) guideline (S1 Checklist)."

The STROBE guideline can be found here: <http://www.equator-network.org/reporting-guidelines/strobe/>

When completing the checklist, please use section and paragraph numbers, rather than page numbers.

We ensured that the study was reported according to the STROBE-nut guideline, included the completed STROBE-nut checklist as Supporting Information and added a statement to the Methods section, p.5, l.137: “All methods have been described in line with the Strengthening the Reporting of Observational Studies in nutritional Epidemiology guidelines (S1 STROBE-nut Checklist)”.

Did your study have a prospective protocol or analysis plan? Please state this (either way) early in the Methods section.

a) If a prospective analysis plan (from your funding proposal, IRB or other ethics committee submission, study protocol, or other planning document written before analyzing the data) was used in designing the study, please include the relevant prospectively written document with your revised manuscript as a Supporting Information file to be published alongside your study, and cite it in the Methods section. A legend for this file should be included at the end of your manuscript.

b) If no such document exists, please make sure that the Methods section transparently describes when analyses were planned, and when/why any data-driven changes to analyses took place.

c) In either case, changes in the analysis-- including those made in response to peer review comments-- should be identified as such in the Methods section of the paper, with rationale.

Details have been added in the Methods section, p.5, l.140, as follows: “The NutriNet-Santé study has been developed to investigate the relationships between multiple dietary exposures and the incidence of chronic diseases, such as cancer, using a prospective approach. The general protocol of the cohort, written in 2008 before the beginning of the study, is available online (<https://info.etude-nutrinet-sante.fr/siteinfo/>). Regarding food additives specifically, the present work is part of a series of pre-specified analyses that are included in a project funded by the European Research Council (ERC, <https://erc.europa.eu/news-events/magazine/erc-2019-consolidator-grants-examples#ADDITIVES>).”

And p.8, l.277, as follows: “The use of Cox models, the three-category coding for sweetener exposure, the adjustment for main confounders (socio-demographic, anthropometric, nutritional and health-related) were pre-specified. The main analyses added following the reviewing process were: linearity check in relation to the trend across categories; interaction tests between artificial sweetener exposure and menopausal status; heterogeneity tests to compare exposure coding strategies; models restricted to non-smoker participants; and analyses exploring the multi-exposure to several types of sweeteners.”

In Figure 3, please ensure that the y axis is identical for all panels to facilitate comparison.

As recommended by Reviewer #2 we have now removed the linearity check using the Restricted Cubic Spline method from the paper and rather checked linearity by comparing the model with the three distinct categories of sweetener intake to a model with a linear trend across these categories, using the Akaike Information Criterion. This was added to the Methods section, p.7, l.228 and the Results section, p.9, l.325: “The comparison of the model with 3 categories of intake to a model with a linear trend across categories did not provide evidence of non-linearity (P=0.107; 0;250; 0.348 and 0.437 for total artificial sweetener; aspartame; acesulfame-K and sucralose, respectively, for the overall cancer model)”.

Throughout, please report P values to 3 decimal places (anything smaller please report as P < 0.001).

P-values were reported with 3 decimals throughout the manuscript and supplemental materials.

Please remove subheadings from your Discussion.

Subheadings have been removed from the Discussion.

Please begin the Discussion with a short, clear summary of the article's findings.

The first paragraph of the Discussion section p.9, l.345 now reads:

“Results from this large-scale prospective cohort study suggest a positive association between higher intake of artificial sweeteners (especially aspartame and acesulfame-K) and overall cancer risk. More specifically, aspartame intake was associated with increased breast and obesity-related cancers.”

Please remove the Competing interest statement, Contributorship statement and guarantor, Transparency statement, Copyright/license for publication statement (all PLOS content is published under a CC-BY license), Data sharing statement, Funding statement and statement of independence of researchers from funders, and LARC disclaimer from the main text. If published, all of this information will be captured as metadata based on your responses to the submission form.

All these statements have been removed from the manuscript and placed in the online submission form.

Please relocate the Patient involvement statement to the Methods section.

The Patient involvement statement has been relocated to the Methods section, p.5, l.147.

**Comments from the reviewers:**

**Reviewer #1**: The Nutri Net-Santé study provides rare data with which to examine the association between artificial sweeteners and health outcomes. It is also a very large data base as required for such work.

We would like to sincerely thank Reviewer #1 for these positive comments and inputs.

Line 29, 'contrasted' seems the wrong word here.

The sentence p.2, l.29 now reads: “The safety of these food additives is debated, with conflicting findings regarding their role in the etiology of various diseases.”

Line 49, remove 'references'.

The sentence p.2, l.53 now reads: “thousands of food and beverage brands”.

Line 58, not well written, maybe '…liking for sweet taste is widespread globally…'

The sentence p.4, l;81 now reads: “liking for sweet taste is widespread globally”.

Line 65, 360 why do you have 'references' after food? Would 'food products' be correct?

Sentences p.2, l.53 and p.4, l.87 now read: “1,400 different food products on the French market” and “thousands of food and beverage brands worldwide”.

Line 68, 'saccharose' is same as sucrose but I would have thought sucrose was better known so would use this term.

The term “sucrose” is now used throughout the manuscript.

In Supplementary Figure 2 'Schoenfeld' is spelt correctly in the heading but not within the figure.

Sorry for the misspelt, it has been corrected throughout the Supplemental document.

**Reviewer #2**: Alex McConnachie, Statistical Review

The paper by Debras et al presents analyses of a large, prospective cohort study, looking at the association between dietary intake of artificial sweeteners and the incidence of cancers. This review considers the statistical parts of the paper.

In short, the statistics in this paper are very good. The background and data sources are well described. The exclusions due to apparent under-reporting are justified. The exclusion of early events to protect against reverse causation is good. Cox models are appropriate, and the key proportional hazards assumption is checked. Models are fully adjusted for a range of potential confounders, and multiple imputation is used to account for missing baseline data. Competing risks models are considered as one of several sensitivity analyses. The results are clearly presented.

The few comments that I do have are fairly minor.

We sincerely thank Reviewer #2 for this overall positive comment. Please find below our responses with additional information and clarifications.

The authors use cubic splines to check linearity. This would be fine, if the main analysis used consumption as a continuous variable, but that is not the case. Each exposure is modelled as a 3-level categorical term (no intake, low intake, high intake), and the significance is tested as a linear trend across categories. It is probably possible for there to exist a linear association between risk and intake as a continuous variable, but a non-linear association between risk and the three categories of intake. There are a few options that I can see: the authors could remove the linearity check from the paper; they could present the HR for each intake as a linear variable (possibly just in the supplements), in which case the linearity check would be relevant; or they could assess linearity in relation to the trend across categories, by comparing the model with 3 categories of intake to a model with a linear trend across categories.

As explained in our response to the Editor’s comment above: *“As recommended by Reviewer #2 we have now removed the linearity check using the Restricted Cubic Spline method from the paper and rather checked linearity by comparing the model with the three distinct categories of sweetener intake to a model with a linear trend across these categories, using the Akaike Information Criterion. This was added to the Methods section, p.7, l.228 and the Results section, p.9, l.325: “The comparison of the model with 3 categories of intake to a model with a linear trend across categories did not provide evidence of non-linearity (P=0.107; 0;250; 0.348 and 0.437 for total artificial sweetener; aspartame; acesulfame-K and sucralose, respectively, for the overall cancer model)”.*

The adjustments in the models are comprehensive, which is good. In similar papers that I have read, it is common to report the unadjusted association, followed by the associations after increasing levels of adjustment for groups of potential confounders. Forest plots can be a good way to present these associations. I would not consider this essential, but it is sometimes interesting to see the size of the unadjusted association, and the extent to which it is confounded by other things, and then which types of factors contribute most to this confounding. Whilst unlikely, it could even be the case that without adjustment, artificial sweetener intake is not associated with outcomes, and the association is only apparent after adjustment.

As recommended, we now report minimally adjusted (for age and sex only) and fully adjusted hazard ratios for the associations between artificial sweeteners (total, aspartame, acesulfame-K, sucralose) and cancer risk (overall, breast, prostate, obesity-related) in Supplementary Figure 2 (forest plots). Overall, the associations were similar between the minimally and the fully adjusted models. This has been added to the Methods p.7, l.246 and Results p.9, l.321 sections.

Line 263-265 states "Analyses conducted among pre- and postmenopausal women distinctively showed that the associations with breast cancers tended to be more pronounced in postmenopausal women." Looking at table S3, I am not convinced. Taking total intakes, the estimated HRs in premenopausal women are 1.21 and 1.24; in postmenopausal women, they are 1.19 and 1.30 - i.e. they are quite similar. The fact that these associations are statistically significant in postmenopausal women only is neither here nor there - I should think that there is no real evidence that the HRs are any different. Any conclusion about different levels of association should be based on statistical interaction tests.

We thank Reviewer 2 for this relevant input. We have detailed this interaction test in the Methods p.7, l.256: “Interactions between baseline menopausal status (menopausal/non-menopausal) and each artificial sweetener among women.” and Results section p.8, l.317: “Associations tended to be more pronounced in postmenopausal women compared to pre-menopausal participants (P for interaction = 0.044; 0.004; 0.033 and 0.152 for total artificial sweeteners; aspartame; acesulfame-k and sucralose, respectively) but all were non-significant due to reduced statistical power (Supplementary Table 3).”

Line 273, the HR for sugar intake is reported as "1." - I reckon this should be about 1.17.

Thank you for this correction, this omission has been corrected p.9, l.334.

Is this in a model that accounts for artificial sweetener intake? This finding is interesting - does it mean that the risk of cancer is roughly the same if someone replaces their sugar intake with artificial sweeteners? How does that affect the conclusion that artificial sweeteners are not a safe alternative to sugar? If the risk of cancer is about the same, but the risk of metabolic conditions is reduced (if that is the case), then maybe they are a safer alternative?

The HR of the association between sugar intake and cancer risk presented p.9, l.333 is a reminder from a previous publication [1]. It was not adjusted for artificial sweetener intakes since the data was not available at the time. However, in the present study, in order to explore the question of which, between sugar or artificial sweeteners, may be the most problematic regarding cancer risk, participants were categorized into 6 classes according to their intake levels of artificial sweeteners (non-consumers, lower and higher) and sugar (below or above the French recommendation of 100 g/d [2]). Cancer risks were compared across the six categories, and in particular between the categories “Higher artificial sweetener consumption & sugar intakes below the official recommended level” and “No artificial sweetener consumption & sugar intakes exceeding the recommended level=reference” (Methods, p.7, l.252). This analysis revealed increased cancer risk associated with both artificial sweetener and sugar intakes (Results, p.9, l.335). The full figure (3-dimensional histogram) corresponding to this analysis is now presented as Supplementary Figure 3, followed by a table providing P-values for the comparison of categories two by two.

Besides, regarding cardiometabolic risks, several observational studies have suggested associations between artificially sweetened beverages and different metabolic conditions [3–8] and artificial sweeteners have also been suggested to increase inflammation [9], alter the gut microbiota [10] and induce glucose intolerance and hyperglycemia. This is why, at this stage of knowledge, several health agencies do not recommend ASB as safe alternative to sugar-sweetened beverages [2]”.

Obviously not for this paper, but are there genetic markers for how much of a "sweet tooth" someone has? Do these factors predict both sugar and artificial sweetener intake? Could a polygenic score be used as part of a Mendelian Randomisation study, to assess the causal association between cancer incidence and the intake of artificial sweeteners (and sugar)?

Thank you for this very interesting question and insights. It appears indeed that genetics could play a role in humans preferences and tastes [11]. For instance, it has been suggested by Soberg et al. that the hepatokine FGF21 (fibroblast growth factor 21) could be linked with sweet taste preference in humans. Subjects with rs838133 variant had lower FGF21 levels and increased sweet consumption. As stated by the authors “investigation of the diet composition in Fgf21 transgenic mice and cynomolgus monkeys revealed that FGF21 reduces appetite for sugars and artificial sweeteners”. This genetic marker, along with potential others, may be integrated into a polygenic score that could be used as part of a Mendelian Randomisation study to assess the causal association between cancer incidence and the intake of artificial sweeteners and sugar. This point has been added in the Discussion section, p.11, l.429.

**Reviewer #3**: Artificial sweeteners and cancer risk in the prospective NutriNet-Sante cohort

In this paper, the authors examined the association of intake of artificial sweeteners and risk of cancer (both overall and site specific) in approximately 100k participants from France. They find that intake of artificial sweeteners is positively associated with risk of cancer, especially breast cancer. Aspartame and acesulfame-K were each individually associated with increased risk of cancer.

This study is clearly scientifically important. Artificial sweeteners, including aspartame and acesulfame-K, are present in thousands of foods and consumed by millions of people. Any association with cancer risk would be scientifically important. The study also addresses a clear gap in the literature. Few epidemiologic studies have examined the association of artificial sweeteners with risk of cancer, particularly breast cancer. Few cohorts have even meaningfully measured or quantified artificial sweetener intake.

A major strength of the current study is that the authors had previously quantified levels of artificial sweeteners in commonly consumed foods and beverages in France. This allows the present study to link its dietary measures to a food database with detailed measures of artificial sweetener composition, providing a superior and more granular measure of artificial sweetener intake than other cohorts have. The primary limitations are that this is only one study of one population and the sample size is not especially large for cancer epidemiology (which is why only a few specific types of cancer could be examined). Studies in cohorts like EPIC or the AARP or in research consortia often have many hundreds of thousands or millions of participants. With additional studies, it remains possible that these associations will converge toward the null in the aggregate, such as occurs with "Winner's Curse". To the authors' credit, they appropriately acknowledge their study's limitations. My recommendations for revisions are described below.

We sincerely thank Reviewer #3 for this positive comment and his/her interesting inputs. Please find below our responses with additional information and clarifications.

**Specific comments**

Abstract background: Artificial sweeteners were originally marketed for weight loss, not for prevention of chronic disease (and excess weight, in of itself, is not a chronic disease). In fact, sodas like Tab, introduced in 1963, pre-date much of the epidemiological evidence on sugar and chronic disease. Consequently, the cause-and-effect sequence described in the first sentence seems somewhat inaccurate.

We thank the Reviewer for this relevant observation, the sentence in abstract background, p.2, l.27, has been modified and now reads: “The food industry uses artificial sweeteners in a wide range of foods and beverages, as alternatives to added sugars for which deleterious effects on several chronic diseases are now well established”.

Abstract background: "findings remain contrasted" should be rewritten as "findings conflict"

The sentence p.2, l;29 now reads: “The safety of these food additives is debated, with conflicting findings regarding their role in the etiology of various diseases.”

Introduction: Some parts of this text need light editing to conform with typical English usage, e.g. "even though these" should be replaced with "that", "expertises" should be "evaluations".

The sentences p.4, l.84 and p.4, l.102 now read:

“manufacturers include artificial sweeteners in some food products that do not traditionally contain added sugar” and “all previous evaluations agreed upon the fact that additional studies, especially in humans, were needed”.

An English native speaker has reviewed the manuscript and modifications have been made throughout the text.

Cancer case ascertainment: Are there any methodological publications documenting the sensitivity and specificity of case ascertainment for the cohort? What percent of cancer cases are ascertained given the relatively recent date of January 22nd, 2021? Could there be a lag in reporting for some cases? If a publication is available, please include a citation here.

No specific methodological paper has been published to document the case ascertainment in the cohort, but additional information has been previously provided [12]. Thus, we have now elaborated the case ascertainment and validation strategy in the Methods section, p.6, l.192: “In order to validate reported incident cancer cases, participants were contacted by a physician of the research team to provide any relevant medical and anatomopathological reports. If necessary, the participant’s physicians and/or hospitals were also contacted to provide the requested information. All cases reported until January 22^nd^ 2021 have been investigated. In addition, the data are linked to the medico-administrative databases of the national health insurance system database (SNIIRAM) and the national mortality registry (CépiDC) to ensure completeness of morbidity and mortality information and to limit bias associated with unreported cases. Medical information was obtained for more than 90% of incident cases and 95% of these were validated, therefore, all incident cases declared were included in the present study, unless they were not validated based on information provided.”

Statistical analysis: Please describe the rationale for reverse causation. Is the idea that some participants get sick with subclinical cancer, change their dietary habits, and then are diagnosed? Or is the concern perhaps more accurately described as recall bias?

We have clarified this point p.8, l.273: “Some participants with subclinical cancer may get sick and change their dietary habits during the months preceding their diagnosis. Thus, in order to challenge this potential reverse causality bias, we performed a sensitivity analysis with follow-up starting at age at entry into the cohort plus two years.”

Statistical analysis: Given that the first two years of cases are excluded, the authors should also state that the first two years of follow-up were also excluded to prevent "immortality bias", since no cancers could occur during this time (i.e. follow-up starts at age at entry into cohort plus two years).

Only cases diagnosed during the first two years (not all subjects) were initially excluded. The requested modification has been made: now, the follow-up starts at age at entry into the cohort plus two years for all participants in the sensitivity analysis aiming to explore potential reverse causality bias (Supplementary Table 6, model 11). In order to limit potential selection bias, the main model is performed on the full sample (Table 2). Both provide similar results. We have modified the Statistical analysis paragraph accordingly, p.8, l.273, as well as results in the Tables and throughout the text.

Results, Supplementary Figure 3: The shaded areas do not appear proportionate to the percentages. If they are not proportionate, consider redrawing or presenting as a table.

The Figure has been turned into a table to represent the number of participants (n, %) in the different categories of artificial sweetener consumption (non-consumers; consumers of aspartame, acesulfame-K or sucralose only, consumers of two types of artificial sweeteners; consumers of aspartame, acesulfame-K and sucralose). This has been modified in the Results section, p.8, p.302, and in Supplementary Table 1.

Results. Associations: Some of the results are hard to read, e.g. breast cancer (HR=1.25 (1.02 to 1.53) P=0.01, HR=1.33 (1.05 to 1.69) P=0.007…respectively). Consider changing use of punctuation to simplify readability.

The sentence p.8, l.313 now reads: “Increased risks were observed for breast cancer (HR=1.22 (1.01 to 1.48) P=0.036 for aspartame) and obesity-related cancers (HR=1.13 (1.00 to 1.28) P=0.036 for artificial sweeteners and, HR=1.15 (1.01 to 1.32) P=0.026 for aspartame).”

Results, Associations: "Associations were consistent in the non-consumers vs. consumers (Supplementary Table 1)". This should include a formal test for heterogeneity.

Models coded in 2 or 3 categories of artificial sweetener exposure were compared with the Akaike Information Criterion. We used the “-2 LOG L” values obtained in the SAS output for both models, subtracted these values and obtained P-value with a χ^2^ test. All P were non-significant, suggesting similar results between the two model types. We added this information in the Methods, p.7, l.231 “Consumers (overall) versus non-consumers analyses were also tested, including a formal test for heterogeneity based on the Akaike Criterion” and Results, p.9, l.324 “Associations were consistent in the non-consumers vs. consumers analyses (Supplementary Table 3) and heterogeneity tests performed revealed no difference between the two model types (all P>0.05)”.

Results, Associations: Was there any consideration given to presenting results for artificially sweetened beverages (ASB)? The results of the current study, at a superficial level, seem to differ from those of the prior BMJ study from this cohort. It would be interesting to know if results differ because of improved measurement in the current study or if measurements of ASB are simply inadequate to characterize artificial sweetener exposure.

Indeed, previous analyses from NutriNet-Santé did not detect an association between ASB and cancer risk [13]. However, in this previous study, exposure to artificial sweeteners was only estimated by using the proxy of the consumption of ASB (i.e. diet soft drinks, sugar-free syrup, and diet milk-based beverages). With improved measurement of artificial sweetener exposure in the present study (from all dietary sources including table-top sweeteners and yogurt/cottage cheese), we were able to detect a significant association between artificial sweetener intake and cancer risk. This suggest that ASB alone is probably insufficient to characterize the overall exposure to artificial sweeteners. We have added discussion about this aspect, p.9, l.364.

Table 1: please clarify what education "no" means. And the other education categories as well. Change "Education level" to "Education level, %". Modify other headings as needed to match.

We apologize for these typos. We have changed the name of this category to “Educational level (%)” and classes to “Less than high school degree”, “2 years or less after high school” and ‘More than 2 years after high school”. We modified other headings as requested.

Table 2: "(mg/d)" is not needed in this table except in the footnote.

Table 2 and Supplementary Tables have been modified accordingly.

Discussion, Mechanistic Plausibility: Reference 12 is a commentary and should not be cited as if it were an independent scientific study. Please also soften the tone of the phrase "recently confirmed in rats". The word "confirmed" implies multiple independent replications, but this is not what was done here. These additional data support, but do not confirm, the original findings.

The commentary reference 12 has been replaced (p.10, l.391) by the scientific study by Tibaldi et al. [14] which completed previous data from the Ramazzini Institute for analysis, classification and diagnosis of lymphoid tissue tumors. The sentence p.10, l.390 has been modified and now reads “Although these findings have been controversial [54] additional data has been recently published, supporting original findings from the Ramazzini Institute, regarding the identification of tumors [55]. This suggests the need for an updated evaluation of aspartame’s carcinogenicity and acceptable daily intake.”

References: Please update ref. 29 now that it is published.

Reference 29 has been updated to:

“Chazelas E, Druesne-Pecollo N, Esseddik Y, Szabo de Edelenyi F, Agaësse C, De Sa A, et al. Exposure to food additive mixtures in 106,000 French adults from the NutriNet-Santé cohort. Sci rep. 2021;11: 19680. doi:10.1038/s41598-021-98496-6”

**Reviewer #4**: Manuscript: Artificial sweeteners and cancer risk in the prospective NutriNet-Santé cohort

Manuscript # PMEDICINE-D-21-04643R1

**General Comments**

This manuscript examined the association of total and specific types of artificial sweetener consumption and cancer risk in a large web-based prospective cohort. Higher intake was associated with higher overall cancer, as well as breast and obesity-related cancers. This manuscript is well-written and contributes to the limited evidence on artificial sweeteners and cancer risk. Only a few minor comments are listed below.

We sincerely thank Reviewer #4 for this positive comment.

**Specific Comments**

1. Results: Although the authors state that the number of high artificial sweetener consumers who consumed all three main types was low and that no interaction was detected between the three types, there are a notable number of participants who consumed at least 2 different types of sweetener. Food products also frequently contain more than one type of artificial sweetener. Can the authors explain whether they tried to investigate this further? This does not necessarily need to be added to the manuscript, but it would be interesting to understand whether the authors considered this since many individuals consumed more than one type of artificial sweetener.

We thank Reviewer 4 for this question. In addition to the analysis of total artificial sweetener intake (summing all types of sweeteners together) and to the test of the 3-way interaction between each main artificial sweetener (aspartame, acesulfame-K and sucralose, no interaction detected), we have performed a new analysis to explore this aspect of multi-sweetener exposure. We classified artificial sweetener consumers into the following categories: consumers of one type of artificial sweetener; consumers of two different sweeteners; consumers of three different sweeteners and compared overall cancer risk two-by-two across these categories, adjusting for the total dose of artificial sweetener intake. This has been added in the Methods p.7, l.263 and Results section p.9, l.327 “After adjustment for the total dose of artificial sweetener exposure, cancer risk did not differ between participants consuming one, two or three different sweeteners (P-values>0.05 for all two-by-two comparisons).”.

For information, in the framework of the ERC-funded project “ADDITIVES” (to which the present work relates), we have identified the main additive mixtures to which participants are exposed [15] (including artificial sweeteners but also other types of additives). The ultimate objective of the project in the next years will be to investigate the relationships between usual exposure to these additive mixtures and chronic disease risk, combining epidemiological and experimental approaches.

2. Supplementary Table 2: It is commendable that the authors examined the intake of artificial sweeteners and sugar, as these may or may not be consumed together. Did the authors also compare additional groups, such as "No artificial sweetener and no sugar" and "High artificial sweetener and high sugar," etc. with the two groups included in this table?

Yes indeed. As explained in our response to Reviewer 2: *“In the present study, in order to explore the question of which, between sugar or artificial sweeteners, may be the most problematic regarding cancer risk, participants were categorized into 6 classes according to their intake levels of artificial sweeteners (non-consumers, lower and higher) and sugar (below or above the French recommendation of 100 g/d* [2]*). Cancer risks were compared across the six categories (Methods, p.7, l.252). This analysis revealed increased cancer risk associated with both artificial sweetener and sugar intakes (Results, p.9, l.333). The full figure (3-dimensional histogram) corresponding to this analysis is now presented as Supplementary Figure 3, followed by a table providing P-values for the comparison of categories two by two.”*

**Supplementary Figure 3**: Cancer risk associated with the combined exposure to artificial sweetener and sugar intakes, NutriNet -Santé cohort, France, 2009-2021 (n=102,865)

3. Discussion, line 292 and 293: It appears that the abbreviation for Cancer Prevention Study-II should be CPS-II.

Sorry for the misspelt, the acronym has been corrected to CPS-II, p.9, l.356.

4. Discussion: It seems that the reason the authors examined the association of artificial sweeteners and obesity-related cancers was because of a potential link between artificial sweeteners and obesity. The manuscript would be strengthened with additional discussion about this.

On the one hand, obesity is a recognized risk factor for many cancer locations: colorectal, stomach, liver, mouth, pharynx, larynx, oesophageal, breast (postmenopause), ovarian, endometrial and prostate cancers [16]. On the other hand, although it remains unclear, associations between artificial sweetener intake and weight gain have been suggested [5,17–21]. Thus, we investigated the associations between artificial sweetener intake and the risk of obesity-related cancers (i.e. cancers locations for which obesity is involved in their aetiology as one of the risk or protective factors). The positive associations observed suggest that part of this relationship may be driven by overweight-related metabolic disturbances. However, the associations between artificial sweetener intake and cancer risk observed in this study are not entirely explained by weight gain-related mechanisms, since the models were adjusted for baseline BMI and weight gain during follow-up. Other mechanisms could be involved, such as inflammation [22] or modification of gut microbiota [10]. We have added some discussion on this aspect, p.10, l.378.

**References**

1.	Debras C, Chazelas E, Srour B, Kesse-Guyot E, Julia C, Zelek L, et al. Total and added sugar intakes, sugar types, and cancer risk: results from the prospective NutriNet-Santé cohort. Am J Clin Nutr. 2020 [cited 18 Sep 2020]. doi:10.1093/ajcn/nqaa246

2.	Anses. Actualisation des repères du PNNS : établissement de recommandations d’apport en sucres. Maisons-Alfort: Anses; 2016 Dec p. 96.

3.	Chazelas E, Debras C, Srour B, Fezeu LK, Julia C, Hercberg S, et al. Sugary Drinks, Artificially-Sweetened Beverages, and Cardiovascular Disease in the NutriNet-Santé Cohort. J Am Coll Cardiol. 2020;76: 2175–2177. doi:10.1016/j.jacc.2020.08.075

4.	Meng Y, Li S, Khan J, Dai Z, Li C, Hu X, et al. Sugar- and Artificially Sweetened Beverages Consumption Linked to Type 2 Diabetes, Cardiovascular Diseases, and All-Cause Mortality: A Systematic Review and Dose-Response Meta-Analysis of Prospective Cohort Studies. Nutrients. 2021;13: 2636. doi:10.3390/nu13082636

5.	Qin P, Li Q, Zhao Y, Chen Q, Sun X, Liu Y, et al. Sugar and artificially sweetened beverages and risk of obesity, type 2 diabetes mellitus, hypertension, and all-cause mortality: a dose-response meta-analysis of prospective cohort studies. Eur J Epidemiol. 2020;35: 655–671. doi:10.1007/s10654-020-00655-y

6.	Yin J, Zhu Y, Malik V, Li X, Peng X, Zhang FF, et al. Intake of Sugar-Sweetened and Low-Calorie Sweetened Beverages and Risk of Cardiovascular Disease: A Meta-Analysis and Systematic Review. Adv Nutr. 2021;12: 89–101. doi:10.1093/advances/nmaa084

7.	Jiang YW, Zhang YB, Pan A. [Consumption of sugar-sweetened beverages and artificially sweetened beverages and risk of cardiovascular disease: a meta-analysis]. Zhonghua Yu Fang Yi Xue Za Zhi. 2021;55: 1159–1167. doi:10.3760/cma.j.cn112150-20210729-00726

8.	Souza B da SN, Cunha DB, Pereira RA, Sichieri R. Soft drink consumption, mainly diet ones, is associated with increased blood pressure in adolescents. J Hypertens. 2016;34: 221–225. doi:10.1097/HJH.0000000000000800

9.	Basson AR, Rodriguez-Palacios A, Cominelli F. Artificial Sweeteners: History and New Concepts on Inflammation. Front Nutr. 2021;8: 746247. doi:10.3389/fnut.2021.746247

10.	Suez J, Korem T, Zeevi D, Zilberman-Schapira G, Thaiss CA, Maza O, et al. Artificial sweeteners induce glucose intolerance by altering the gut microbiota. Nature. 2014;514: 181–186. doi:10.1038/nature13793

11.	Søberg S, Sandholt CH, Jespersen NZ, Toft U, Madsen AL, von Holstein-Rathlou S, et al. FGF21 Is a Sugar-Induced Hormone Associated with Sweet Intake and Preference in Humans. Cell Metab. 2017;25: 1045–1053. doi:10.1016/j.cmet.2017.04.009

12.	Fiolet T, Srour B, Sellem L, Kesse-Guyot E, Allès B, Méjean C, et al. Consumption of ultra-processed foods and cancer risk: results from NutriNet-Santé prospective cohort. BMJ. 2018; k322. doi:10.1136/bmj.k322

13.	Chazelas E, Srour B, Desmetz E, Kesse-Guyot E, Julia C, Deschamps V, et al. Sugary drink consumption and risk of cancer: results from NutriNet-Santé prospective cohort. BMJ. 2019;366: l2408. doi:10.1136/bmj.l2408

14.	Tibaldi E, Gnudi F, Panzacchi S, Mandrioli D, Vornoli A, Manservigi M, et al. Identification of aspartame-induced haematopoietic and lymphoid tumours in rats after lifetime treatment. Acta Histochem. 2020;122: 151548. doi:10.1016/j.acthis.2020.151548

15.	Chazelas E, Druesne-Pecollo N, Esseddik Y, Szabo de Edelenyi F, Agaësse C, De Sa A, et al. Exposure to food additive mixtures in 106,000 French adults from the NutriNet-Santé cohort. Sci Rep. 2021;11: 19680. doi:10.1038/s41598-021-98496-6

16.	WCRF/AICR. Continuous Update Project Expert Report 2018. Recommendations and public health and policy implications. 2018; 92.

17.	Azad MB, Abou-Setta AM, Chauhan BF, Rabbani R, Lys J, Copstein L, et al. Nonnutritive sweeteners and cardiometabolic health: a systematic review and meta-analysis of randomized controlled trials and prospective cohort studies. CMAJ. 2017;189: E929–E939. doi:10.1503/cmaj.161390

18.	Miller PE, Perez V. Low-calorie sweeteners and body weight and composition: a meta-analysis of randomized controlled trials and prospective cohort studies123. Am J Clin Nutr. 2014;100: 765–777. doi:10.3945/ajcn.113.082826

19.	Pang MD, Goossens GH, Blaak EE. The Impact of Artificial Sweeteners on Body Weight Control and Glucose Homeostasis. Front Nutr. 2021;7. doi:10.3389/fnut.2020.598340

20.	Davidson TL, Martin AA, Clark K, Swithers SE. Intake of High-intensity Sweeteners alters the Ability of Sweet Taste to Signal Caloric Consequences: Implications for the Learned Control of Energy and Body Weight Regulation. Q J Exp Psychol 2006. 2011;64: 1430–1441. doi:10.1080/17470218.2011.552729

21.	Feijó F de M, Ballard CR, Foletto KC, Batista BAM, Neves AM, Ribeiro MFM, et al. Saccharin and aspartame, compared with sucrose, induce greater weight gain in adult Wistar rats, at similar total caloric intake levels. Appetite. 2013;60: 203–207. doi:10.1016/j.appet.2012.10.009

22.	Alleva R, Borghi B, Santarelli L, Strafella E, Carbonari D, Bracci M, et al. In vitro effect of aspartame in angiogenesis induction. Toxicol In Vitro. 2011;25: 286–293. doi:10.1016/j.tiv.2010.09.002
