# Supplementary material for: Artificial sweeteners and cancer risk: Results from the NutriNet-Santé population-based cohort study
Source: PLoS Med. 2022 Mar 24;19(3):e1003950. doi: 10.1371/journal.pmed.1003950 (PMC8946744; doi:10.1371/journal.pmed.1003950)
Supplement: Attachment — Submitted filename: AUTHOR RESPONSE LETTER_PlosMed_R2.docx [file pmed.1003950.s004.docx]

Author Response Letter – Revision 2

**Point-by-point Response**

We would like to sincerely thank the Editors and the Reviewers for granting us the opportunity to further revise this paper. Please find below our point-by-point responses.

The references to page/line numbers correspond to the new clean version of the manuscript.

We also went through the **Guidelines for revised papers** and made the requested edits throughout the manuscript.

**Request from the editors:**

Please update your title to ‘Artificial sweeteners and cancer risk: a population-based cohort study’ or similar (please do not include the word ‘prospective’).

The title has been changed as suggested to “Artificial sweeteners and cancer risk: results from the NutriNet-Santé population-based cohort study” (p.1, l.4).

Lines 49-51: Please shorten the limitations section of your abstract to a single sentence (e.g. ‘Limitations of this study include potential selection bias, residual confounding, and reverse causality, though sensitivity analyses were performed to address these concerns.’ or similar).

The sentence was shortened as suggested and now reads: “Limitations of this study include potential selection bias, residual confounding, and reverse causality, though several sensitivity analyses were performed to address these concerns” (p.2, l.49).

Lines 57-8: Please remove the trial registration number/URL from the abstract.

The trial registration number and the URL have been removed from the abstract.

Line 71: Please quantify the key findings mentioned in your Author Summary (with 95% confidence intervals and p-values).

We have added precisions to the key findings in the Author Summary which now reads (p.3, l.71):

- “In this large cohort of 102,865 French adults, artificial sweeteners (especially aspartame and acesulfame-K) were associated with increased overall cancer risk: hazard ratio for higher consumers compared to non-consumers = 1.13, 95% confidence interval = 1.03 to 1.25, P-trend = 0.002.
- More specifically aspartame intake was associated with increased breast (HR=1.22 (1.01 to 1.48) P=0.036) and obesity-related (HR=1.15 (1.01 to 1.32) P=0.02) cancer risks.”

Thank you for including the STROBE-nut checklist – please update the checklist to use section names/paragraph numbers as opposed to page numbers (which are liable to change during the revision process).

We have modified the STROBE-nut checklist which now displays section names instead of page numbers.

As no pre-specified analysis plan is available for this study, the term ‘prospective’ should be avoided throughout; please refer to the study instead as a population-based cohort study. If the authors wish to maintain the ‘prospective’ descriptor, it would be best if a document detailing the pre-specified analysis plan could be provided in the supporting information (the URL provided on lines 145-6 appears to be a news article).

As requested we have now removed the term “prospective” throughout the text.

Similar to the above, apologies if I have missed it but I could not locate the general protocol at the provided URL (https://info.etude-nutrinet-sante.fr/siteinfo/), would it be possible to provide a direct link to the protocol?

The direct link to the NutriNet-Santé cohort protocol has been added p.5, l.147:

https://info.etude-nutrinet-sante.fr/upload/siteinfo/files/ck/files/protocole%20general%20en%20anglais%20NutriNet_Aug2013_EM_03_09_13.pdf

Please confirm whether SG’s affiliation with Open Food Facts ought to be declared as a COI.

We did not think that it was a COI since Open Food Facts is a non-profit, open data association, but for total transparency we have now added this information in the COI box online:

“*SG is co-founder of Open Food, a non-profit project developed by thousands of volunteers from around the world. It is a free and open-data food product database designed to help citizens make better food choices.*”

Lines 262/332: Please tabulate this data and present it in the supporting information.

We have now tabulated this data, which is presented in Supplementary Table 4 of supporting information and have modified the manuscript accordingly p.7, l.263 and p.9, l.338 as follows:

“Formal interactions between BMI (</≥25 kg/m^2^) and each artificial sweetener were also tested. Similarly, the 3-way interaction between each main artificial sweetener (aspartame, acesulfame-K and sucralose) was tested for all studied outcomes by including the product of the three variables into Cox models”

“No interaction was detected for any cancer outcome between artificial sweetener exposures and BMI, nor between the three main artificial sweeteners (Supplementary Table 4).”

Line 433: Please remove this subheading in the Discussion.

The subheading has been removed.

Please show the unadjusted comparisons as well as the adjusted comparisons in Table 2.

Unadjusted comparisons have been added in Table 2 p.20.

**Comments from the reviewers:**

**Reviewer #2: Alex McConnachie, Statistical Review**

I thank the authors for their consideration of my original points. I feel that the paper has been improved, though I have one remaining comment.

We sincerely thank Reviewer #2 for this overall positive comment. Please find below our responses with additional information and clarifications.

The data presented in supplementary table 2 do not seem to be in line with the interaction p-values reported. For total artificial sweeteners, the interaction p-value is given as 0.044, suggesting different associations between intake and premenopausal breast cancer compared to postmenopausal breast cancer. However, the hazard ratios do not appear to be very different. For low consumers vs. non-consumers, the HRs are the same (1.09), and for high consumers vs. non-consumers, the difference is small (1.15 vs. 1.20) with confidence intervals that entirely overlap. These HRs and CIs do not, in my opinion, seem consistent with an interaction p-value of 0.044. If anything, it is for sucralose, where the associations trend in opposite directions, where I might anticipate a significant interaction, but this interaction is reported as p=0.152.

We thank Pr McConnachie for this relevant comment. You are right. The discrepancy between the HR (95%CI) results from pre/post menopausal models on the one hand and interaction tests on the other hand is explained by the fact that the interaction test was performed by using baseline menopausal status, whereas our stratified analyses accounted for the age at menopause, i.e., for the premenopausal model, women contributed up to their age at menopause at the latest, and for the postmenopausal model, women contributed at the earliest from their age at menopause (thus some women contributed to both models, with different person-time).

Appropriate heterogeneity test has now been performed instead, and the Statistical analysis section has been amended accordingly (p.7, l.260): “Further analyses were conducted to investigate the associations with premenopausal and postmenopausal breast cancers separately and menopause-related heterogeneity was assessed using likelihood ratio tests by comparing the estimated log-likelihood of a model to that of the same model plus a multiplicative interaction term for menopausal status and the artificial sweetener exposure.”.

Results from these heterogeneity tests are now consistent with hazard ratios reported in Supplementary Table 2, as presented in the Results paragraph (p.8, l.332): “Overall the same direction was observed in pre- and postmenopausal women (Supplementary Table 2). Heterogeneity tests showed no difference between pre- and postmenopausal models for total artificial sweeteners, aspartame and acesulfame-K (P for heterogeneity = 0.440, 0.332 and 0.359, respectively). P for heterogeneity was 0.015 for sucralose but associations with cancer risk were non-significant in both strata for this food additive, with a low number of consumers per strata”.

Also, I spotted a couple of typos:

In supplementary table 3, for all cancers, sucralose, the HR is reported as 1.10, which is very close to the upper confidence limit. Should this be 1.01?

Thank you for this correction, the HR was 1.00, it has been corrected in Supplementary Table 3.

In supplementary table 4, the HR between [ No AS / Low sugar ] and [ Low AS / Low sugar ] should be less than 1, but is reported as 1.15, the same as the opposite comparison.

Thank you for this correction, the HR was indeed 0.87 (0.79-0.96) P=0.006 for No AS / Low sugar versus Low AS / Low sugar. We apologize for this typo.

**Reviewer #3: I am satisfied with the revisions made to this article.**

We thank Reviewer 3 for his/her feedback.

**Reviewer #4: The authors have adequately addressed the comments of the Editors and Reviewers.**

Minor editing is needed to correct small errors (e.g., Introduction, lines 82-83- could be edited to "…reduce added sugar content and corresponding calories while maintaining sweetness"; Results, page 9, line 326- comma instead of semicolon for 0.250; Discussion, line 357- "CPS-II" cohort, etc.).

We thank Reviewer 4 for his/her positive comment. Sorry for these typos, we have corrected them throughout the text.
