# Supplementary material for: Artificial sweeteners and cancer risk: Results from the NutriNet-Santé population-based cohort study
Source: PLoS Med. 2022 Mar 24;19(3):e1003950. doi: 10.1371/journal.pmed.1003950 (PMC8946744; doi:10.1371/journal.pmed.1003950)
Supplement: Attachment — Submitted filename: AUTHOR RESPONSE LETTER_PlosMed_R3.docx [file pmed.1003950.s005.docx]

Author Response Letter – Revision 3

**Point-by-point Response**

We would like to sincerely thank the Editors and the Reviewers for granting us the opportunity to further revise this paper. Please find below our point-by-point responses.

The references to page/line numbers correspond to the new clean version of the manuscript.

We also went through the **Guidelines for revised papers** and made the requested edits throughout the manuscript.

**Request from the editors:**

Lines 52, 79, and 443: Please update to ‘many food and beverage brands worldwide’.

This has been modified l.52, 77 and 444.

Line 71: Please add ‘(HR)’.

This has been added l.70.

Figures 2 and 3: The legends for these figures do not explain the asterisks in the figures.

Figure legends p.22 have now been completed and include the asterisk in the figures.

Lines 339-349: Please consider whether some of this content would be better placed in the Discussion (presentation of results from the previous study on lines 339-341 and the interpretation at lines 347-348). Presentation of the data in the Supplementary Materials should however remain in the Results section.

The paragraph has now been modified as follows: presentation of results from the previous study and interpretation have been moved to the Discussion l.377 and presentation of the data in the Supplementary Materials remained in the Results section l.338.

Supplementary Results: I could not find reference to this part of the Supplementary Materials in the manuscript.

Reference to Supplementary Results was added l.327 as follows: “Results for competing risks are presented in Supplementary Results.”

**Comments from Reviewers:**

Reviewer #2: Alex McConnachie, Statistical Review

I thank the authors once again for considering my comments, and I am happy with the modifications they have made. I have no further comments.

We sincerely thank Pr. McConnachie for his feedback.
